# Phosphatidylinositolmannoside vaccination induces lipid-specific Th1-responses and partially protects guinea pigs from *Mycobacterium tuberculosis* challenge

**DOI:** 10.1038/s41598-023-45898-3

**Published:** 2023-10-30

**Authors:** Emmelie Eckhardt, Jan Schinköthe, Marcel Gischke, Julia Sehl-Ewert, Björn Corleis, Anca Dorhoi, Jens Teifke, Dirk Albrecht, Annemieke Geluk, Martine Gilleron, Max Bastian

**Affiliations:** 1 Friedrich-Loeffler-Institut, Südufer 10, 17493 Greifswald – Isle of Riems, Germany; 2 Institute of Veterinary Pathology, Faculty of Veterinary Medicine, Leipzig University, Leipzig, Germany; 3 Institute of Microbiology, Greifswald University, Greifswald, Germany; 4 Department of Infectious Diseases, Leiden University Medical Center, Leiden, The Netherlands; 5 CNRS, Institut de Pharmacologie et de Biologie Structurale, Toulouse, France

**Keywords:** Tuberculosis, Inactivated vaccines, Immunological memory, Glycolipids, Infection

## Abstract

The concept of donor-unrestricted T cells (DURTs) comprises a heterogeneity of lymphoid cells that respond to an abundance of unconventional epitopes in a non-MHC-restricted manner. Vaccinologists strive to harness this so far underexplored branch of the immune system for new vaccines against tuberculosis. A particular division of DURTs are T cells that recognize their cognate lipid antigen in the context of CD1-molecules. Mycobacteria are characterized by a particular lipid-rich cell wall. Several of these lipids have been shown to be presented to T cells via CD1b-molecules. Guinea pigs functionally express CD1b and are hence an appropriate small animal model to study the role of CD1b-restricted, lipid-specific immune responses. In the current study, guinea pigs were vaccinated with BCG or highly-purified, liposome-formulated phosphatidylinositol-hexa-mannoside (PIM_6_) to assess the effect of CD1-restricted DURTs on the course of infection after virulent *Mycobacterium tuberculosis* (*Mtb*) challenge. Robust PIM_6_-specific T cell-responses were observed both after BCG- and PIM_6_-vaccination. The cellular response was significantly reduced in the presence of monoclonal, CD1b-blocking antibodies, indicating that a predominant part of this reactivity was CD1b-restricted. When animals were challenged with *Mtb*, BCG- and PIM_6_-vaccinated animals showed significantly reduced pathology, smaller necrotic granulomas in lymph node and spleen and reduced bacterial loads. While BCG conferred an almost sterile protection in this setting, compared to control animals’ lesions were reduced roughly by two thirds in PIM_6_-vaccinated. Comprehensive histological and transcriptional analyses in the draining lymph node revealed that protected animals showed reduced transcription-levels of inflammatory cyto- and chemokines and higher levels of CD1b-expression on professional antigen cells compared to controls. Although BCG as a comparator induced by far stronger effects, our observations in the guinea pig model suggest that CD1b-restricted, PIM_6_-reactive DURTs contribute to immune-mediated containment of virulent *Mtb*.

## Introduction

*Bacille Calmette-Guérin* (BCG) is still the only licensed vaccine against tuberculosis (TB). It shows limited efficacy against the epidemiologically most relevant lung manifestation of TB, but it protects reliably from severe systemic TB during early childhood^1^. BCG activates different layers of the immune system. This comprises the induction of adaptive memory and an education of the innate immune system, now known as trained immunity^2^. Many prominent protein antigens are targets of protective adaptive immune responses^3^. However, it is likewise clear that mycobacteria display a broad repertoire of complex lipids and lipoglycans that also provoke adaptive immune responses. In a recent publication, we have shown that BCG-vaccinated guinea pigs respond to lipid-extracts with robust T cell-proliferation^4^. The prominent T cell-responses are in part due to MHC-II-presented, lipophilic peptides that are present in total lipid extracts of cultured mycobacteria^5^. However, CD1-restricted T cell-responses have been reported that are directed against structurally well-defined mycobacterial lipid antigens^6–9^. CD1-molecules are antigen-presenting molecules that are only expressed by professional antigen presenting cells and were evolved to present lipid antigens to T cells. Since CD1-molecules are non-polymorphic, there are no differences in the epitope binding and presentation capacities between different individuals. It is an appealing concept to incorporate such universally binding epitopes as subunit components in future TB-vaccines. For this concept the term donor-unrestricted T cells (DURTs) has been coined. In addition to CD1-restricted T cells, DURT cells comprise cells that recognize their antigen in the context of MHC related protein 1 (MR1), butyrophilin 3A1, as well as the nonclassical MHC class Ib family member HLA-E^10^. Among CD1-restricted T cells there are clear functional differences: NKT cells for example recognize their antigen in the context of the group-II CD1d-molecule. They bear an invariant T cell receptor (TCR) and represent an intermediate between innate NK and adaptive T cells^11^. They contribute to antimicrobial immune defense, but are also known to be involved in autoimmune disorders^12^. By contrast, group-I CD1-restricted T cells that recognize their antigen in humans in the context of CD1a, CD1b or CD1c express variable αβ-TCRs. Their function is only partly understood and there are numerous reports on the recognition of lipid autoantigens, which may contribute to local or general immune homeostasis (see^13^ for a recent overview). At the same time, it is well established that CD1b-restricted T cells can recognize lipids that are specific for certain microbes^6,14^. These cells truly belong to the adaptive compartment of the immune system and require priming to exert their immune function^15^. Of all CD1b-restricted lipids, phosphatidylinositolmannosides (PIMs) are a class of highly glycosylated lipids that form the lipid anchor of lipoarabinomannan in the mycobacterial cell wall^16^. The molecular mechanisms, how lipids are loaded into the hydrophobic binding groove of CD1b-molecules are well studied^17,18^. However, the functional implications of this system are less clear. The development of lipid-loaded CD1b-tetramers was an important milestone for a better understanding of the CD1-T cell-axis. Ex vivo tetramer staining allowed for a phenotypical characterization of CD1a- and CD1b-restricted T cells and revealed that the majority of these cells was CD4-positive^19,20^. A deeper understanding on the functional role of CD1-group-1-restricted T cells in the immune defense against pathogens has long been hampered by the fact that the most common rodent animal model, the mouse, by nature does not express a functional CD1-group-1-system. A well-established small rodent model that has already been used by the early pioneers of TB-research is the guinea pig^21^. Guinea pigs naturally express a broad panel of CD1-group-1-molecules. Founding studies investigated the repertoire of CD1-isoforms and found four CD1b- and three CD1c-isoforms that are functionally expressed in the guinea pig^22,23^. Detailed molecular analyses indicated that the CD1b1- and CD1b4-isoform are functionally relevant in their resemblance to human CD1b-molecules, while CD1b3 is mostly expressed on B cells^24^. Furthermore, in a series of publications it was demonstrated that lipid-vaccinated guinea pigs are at least partially protected from virulent *Mycobacterium tuberculosis* (*Mtb*)-challenge^25–28^. So, the protective effect of lipids in general has been shown, but the precise nature of the immune response triggered has yet to be described. It is unclear, which lipids are presented during mycobacterial infection. It has to be further clarified, which cells respond to CD1b-restricted lipids and where they encounter their antigen. Finally, the effect of CD1-restricted T memory cells on the course of a mycobacterial challenge has to be elucidated. To address these questions and investigate the specific role of CD1-restricted DURT cells, in the current study, we investigated lipid-specific responses in BCG-vaccinated guinea pigs. Based on previous studies^25^ and own unpublished experiments we then vaccinated guinea pigs with liposome-formulated PIM_6_ and investigated the effect on the course of infection after virulent *Mtb-*challenge.

## Material and methods

### Bacteria

BCG, strain Pasteur_1173_, was obtained from Dr. Walter Matheis (Paul-Ehrlich-Institut, Germany). The strain was grown in-house. A working stock was established, frozen at -80 °C and used throughout the study. *Mtb*, strain H37Rv, was kindly provided by Prof. Dr. Stefan H.E. Kaufmann (Max Planck Institute for Infection Biology, Germany). Bacteria were grown in Middlebrook 7H9-Medium (Becton–Dickinson, Germany) enriched with OADC (Becton–Dickinson, Germany) and 0.05% Tween80 (Sigma-Aldrich, Germany).

### Liposomes

The composition and preparation of liposomes corresponded to the Cationic Adjuvant Formulation (CAF01), as described^29^. Briefly, 625 µg dimethyldioctadecylammonium-bromide (DDA, Avanti Polar Lipids) and 125 µg trehalose 6,6′-dibehenate (TDB, Avanti Polar Lipids), solubilized either in chloroform or in chloroform:methanol (9:1), were combined per dose. For PIM_6_-liposomes, 25 µg of a highly-purified PIM_6_-preparation were added. PIM_6_ was purified and characterized by MALDI-TOF-analysis, as described^29^. The components were mixed and exsiccated. Subsequently, lipid films were rehydrated with 0.5 ml of Tris-buffered, distilled water and resolubilized by mild sonification.

### Animal experiments

#### Ethic statement

All animal experiments were approved by the ethics committee of the responsible competent authority, the State Office of Agriculture, Food Safety and Fishery in Mecklenburg-Western Pomerania (LALLF MV, 7221.3–1-065/15). All experiments were conducted in accordance with German and European animal welfare legislation and in compliance with the ARRIVE guidelines.

#### Animal husbandry

Female Dunkin-Hartley guinea pigs were obtained from Charles River Laboratories, Sulzfeld, Germany. Animals were housed in groups of three in plastic-cages on dust-free wooden bedding. They had free access to dry pellets and water. After completion of the study guinea pigs were anesthetized as described below and euthanized by carbon-dioxide inhalation. Death was confirmed during the following dissection.

#### Experimental BCG-vaccination

One group of guinea pigs (n = 8) was administered 1 × 10^6^ CFUs BCG_Pasteur_ resuspended in 0.5 ml saline subcutaneously to the left axillary region. A control group (n = 7) received saline control in parallel. Four weeks after immunization blood was obtained by non-terminal cardiocentesis and processed to analyse cellular immune responses.

#### Experimental PIM_6_-vaccination

Three groups of six guinea pigs were immunized in parallel (n = 18): 1 × 10^6^ CFUs BCG_Pasteur_, PIM_6_-, or empty CAF01-liposomes were resuspended in 0.5 ml saline and administered subcutaneously to the left axillary region. Liposomes were administered three times with an interval of two weeks. Non-terminal cardiocentesis was performed on anesthetized animals before and 28 and 80 days after the first vaccination. Blood was processed to analyse cellular immune responses.

#### Experimental challenge

The same vaccinated guinea pigs (n = 18) were challenged 84 days after the first immunization, by subcutaneous inoculation of 1 × 10^3^ CFUs of *Mtb* in the right axillary region. An additional group of non-immunized animals (n = 6) was likewise infected. Four weeks after challenge the animals were euthanized. Tissue samples were taken from the injection site, the draining right axillary lymph node, the spleen and the lung. They were used for histopathological, cultural and transcriptional analyses. To analyse the transcriptional profile prior to the challenge, an additional vaccination experiment was performed (n = 18).

### Assessment of cellular immune responses

#### Antigens

Phytohaemagglutinin (PHA, Oxoid, Germany) was used as positive control. A total sonicate (Lysate_BCG_) and a chloroform–methanol extract (CME_BCG_) were produced, as described^4^. Protein antigens, Antigen-85-A and ESAT6 were expressed in *E. coli* and purified as described^30^. For lipid-specific stimulation purified mannosylated lipoarabinomannan (LAM, NR-14848); lipomannan (LM, NR-14850); phosphatidylinositol-hexa-mannoside (PIM_6_, NR-14847) and phosphatidylinositol-di-mannoside (PIM_2_, NR-14846) all purified from *Mtb*_H37Rv_, were obtained from BEI Resources, NIAID, NIH. For a better comparison this set of four related antigens was obtained from one source. Phthiocerol Dimycocerosate (PDIM, NR-20328) was also obtained from BEI Resources. Glucose-monomycolate (GMM) was purified, as described^6^.

#### Lymphocyte preparation

Peripheral blood mononuclear cells (PBMCs) were isolated using Ficoll-Paque-gradient-centrifugation, as described^31^. Plastic adherent monocytes were incubated overnight at 37 °C and 5% CO_2_ in Iscove’s-modified-Dulbeco’s-medium (IMDM, in-house) supplemented with 5% autologous serum and 5% conditioned hybridoma supernatant containing guinea pig IL4 and GM-CSF to induce CD1-expression.Non-adherent responder cells were stored overnight.

#### Proliferation assay

After overnight incubation, non-adherent responder cells were mixed with CD1-expressing, autologous antigen presenting cells (APCs) at a 3:1 ratio and stained with carboxyfluorescein-succimidyl-ester (CFSE, Enzo Life Science, Germany), as described^31^. Subsequently, 1.3 × 10^5^ cells per well were seeded in 96-well round-bottom-plates (Greiner Bio-One, Germany) in 100 µl IMDM-medium containing 10% autologous serum. Cells were stimulated in duplicates. Non-stimulated cells served as medium control. CFSE dilution was analysed after 5 days of incubation at 37 °C using a MACS Quant Analyzer (Miltenyi Biotec, Germany).

#### Flow cytometry

CFSE-negative lymphocytes were further characterized by a triple-staining using allophycocyanin-conjugated mouse-anti-guinea-pig-T cell-antibody (AbD Serotec, Germany); phycoerythrin-conjugated mouse-anti-guinea-pig-CD4 antibody (AbD Serotec) and biotinylated mouse-anti-guinea-pig-CD8 antibody (kindly provided by Dr. Hubert Schäfer, Robert Koch-Institut, Germany). Binding of biotinylated anti-CD8 antibody was visualized using PE-Cy5.5-conjugated streptavidin (Invitrogen, Germany). Flow cytometry was performed using a MACS Quant Analyzer (Miltenyi, Germany). FlowJo-Software (Version 9.9.6) was used to analyse flow-cytometric data.

### Pathology

Four weeks after the challenge with *Mtb*, guinea pigs were humanely euthanized. Necropsies were performed under BSL3-conditions. Blinded, macroscopic scoring of gross lesions was performed for the right axillary subcutis, right axillary lymph node, spleen, and liver by assessing formation of granulomas, number and size of granulomas and presence of necrosis. The scores were derived from an ordinally scale of 0–4 based on the modified Mitchison scoring system detailed in Supplemental Table S1^32,33^.

### Measurement of bacterial growth

Tissue samples of the spleen were homogenized in 1 ml of PBS containing 0.05% Tween80 (Sigma-Aldrich, Germany). The homogenate was 1:10 serially diluted to a dilution of 10^–7^. 50 µl of each dilution was plated on 7H11-agar plates (Becton–Dickinson, Germany). Agar plates were incubated at 37 °C for 3 weeks before determining number of CFUs.

### Transcription-analysis

#### Sample preparation

To determine antigen-specific upregulation of cytokine transcripts non-adherent responder cells and autologous APCs were stimulated as described above. For CD1b-blocking experiments cells were stimulated with PIM_6_ in the presence or absence of equivalent amounts of anti-guinea-pig-CD1b- or isotype-matched-hybridoma-supernatant (CD1F2 hybridoma, kindly provided by Steven Porcelli, Albert-Einstein-College; or anti rabies-G-Protein antibody E559, kindly provided by Thomas Mueller, FLI). After 24 h, cells were washed and solubilized in TRIzol Reagent (Fisher Scientific, Germany). Tissue samples from injection-site-granulomas, draining axillary lymph nodes and spleen were collected during necropsy and put into TRIzol. Tissue samples were homogenised using a gentleMACSDissociator (Miltenyi Biotec, Germany).

#### RNA isolation and real-time PCR

RNA was isolated using TRIzol and RNeasy Mini Kit (Qiagen, Germany). For qRT-PCR, QuantiTect SYBR Green PCR Kit (Qiagen, Germany) was used. Primers were designed using the NCBI Primer-BLAST tool (^32^; see Supplemental Table S2). Ct-values were determined and relative transcription-levels were calculated in relation to β-Actin according to the following equation: transcription-level = 1000 × 2^(Ct b-Act – Ct test)^.

### Histological analysis

#### Tissue preparation and staining

Formaldehyde-fixed, paraffin-embedded (FFPE) tissues were cut at 3 µm, and stained with hematoxylin–eosin (HE) for microscopic analysis, according to standard procedures^34^. Injection-site lesions and axillary lymph nodes were available from five CAF01- and PIM_6_-vaccinated animals, from BCG-vaccinated animals one injection-site-granuloma and two lymph nodes were available. Spleen sections were available from all six animals per group. Tissue sections were prepared for immunophenotyping and mRNA-detection as follows.

#### Immunohistochemistry

Mycobacteria, B cells, T cells and macrophages were detected with primary antibodies described in Supplemental Table S3. As secondary antibody a biotinylated goat-anti-mouse-IgG (Vector Laboratories, USA) was used. For visualization of B cells VECTASTAIN ABC Kit (Vector Laboratories) and for mycobacteria, macrophages and T cells the EnVision^+^ System (Dako, USA) was used.

#### In situ hybridisation (ISH)

ISH was performed using the RNAscope 2.5 HD Reagent Kit-RED (ACD biotechne, Germany) according to the manufacturer’s instructions. Specific probes for guinea pig CD1b1, IFNγ and CXCL10 were custom designed and provided by the manufacturer. According to the manufacturer, the CD1b1-probe does not discriminate between CD1b1- and CD1b4-transcripts. Stainings obtained with the CD1b1-probe are therefore considered synonymous for CD1b1 and b4. As controls, DapB- (negative) and Cp-Ppib-probes (positive) were used (see supplemental Figure S5 for control stainings).

#### Whole Slide Image (WSI) analysis

All histological sections obtained after the challenge were scanned using a digital slide scanner (Hamamatsu, Germany). QuPath software^35^ was used to analyse all digitalised images. In some BCG-vaccinated animals, granulomas and lymph nodes were too small to perform histopathological and transcriptional analysis in parallel. In those cases, we prioritized the transcriptional analysis of the expression profiles. For each animal the absolute lesion area in each tissue section of the injection site, lymph node and spleen was determined. Within granulomas, macrophage-rich areas were differentiated from necroses, and the ratio was calculated. In the lymph nodes, regions of interest (ROI) were defined as granuloma and unaltered lymphatic tissue, comprising B- and T cell-areas. In a number of visual fields representative for the size and type of ROI, the number of mycobacteria, macrophages, B-, T cells and CD1- or cytokine-expressing cells were counted automatically at 400×-magnification using the positive cell detection tool of the software.

### Statistics

**Figure 1:**
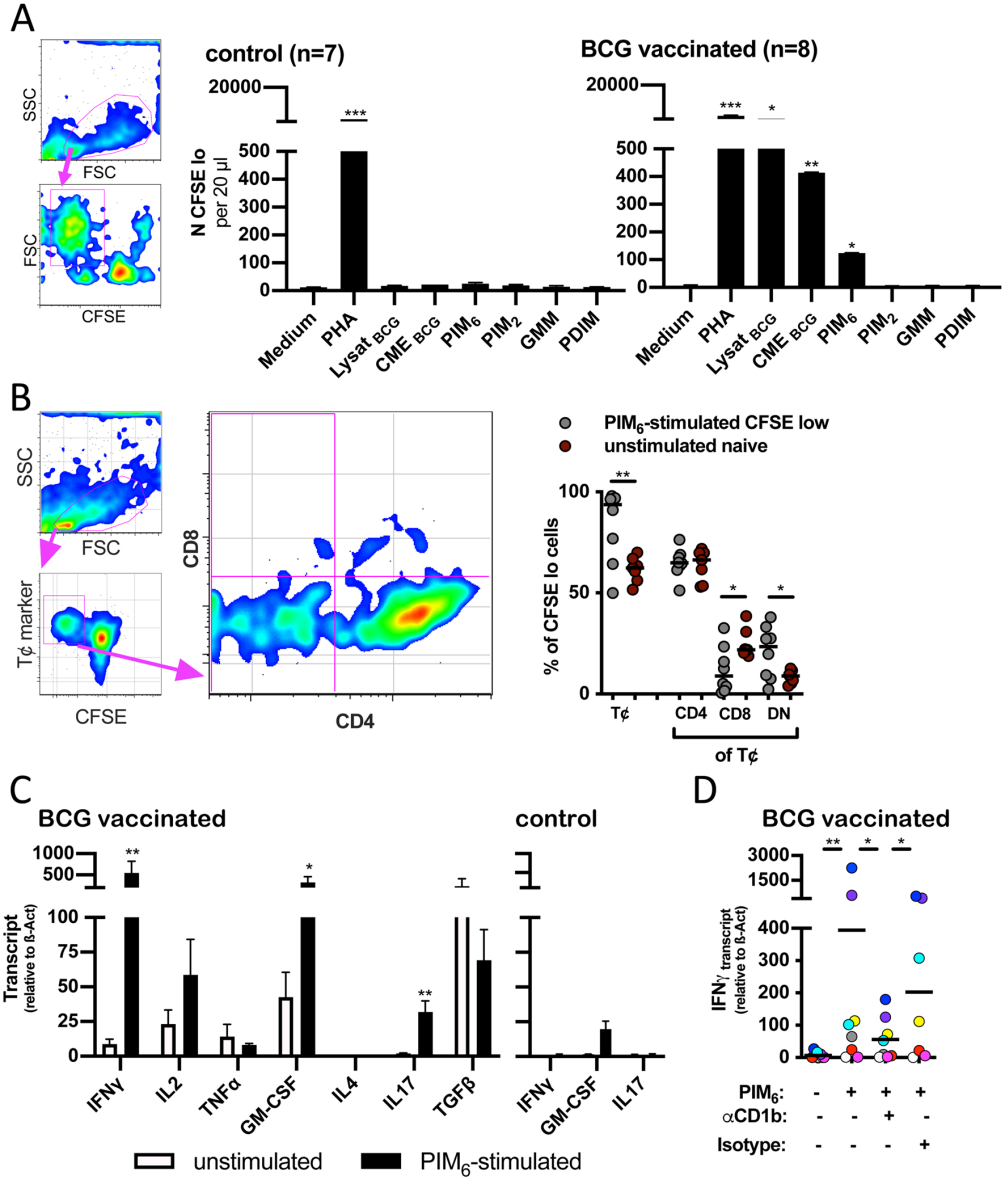
BCG-vaccination elicits CD1b-restricted, PIM_6_-specific T cell-responses. Four weeks after BCG-vaccination PBMCs were isolated. Autologous, CD1b-expressing APCs and non-adherent lymphocytes were stained with CFSE and stimulated with mycobacterial antigens. CFSE dilution was analyzed by flow-cytometry after five days. (**A**) Seven guinea pigs were control-treated. Eight guinea pigs were BCG-vaccinated. After four weeks, T cell-proliferation in response to the indicated mycobacterial antigen was assessed. The graphs on the left depict the gating strategy. In the diagrams, black bars represent the group mean as determined by nested-data-analysis. Error bars represent the standard error of the mean. Asterisks indicate the level of significance as determined by nested-t-test in comparison to the medium control. (**B**) CFSE-low, PIM_6_-reactive lymphocytes were stained for a general T cell-marker, CD4 and CD8. The small panel at the left shows the gating strategy. The larger pseudo-color graph representatively shows the CD4- and CD8-distribution. The dot blot on the right shows the relative distribution for T cell-markers in PIM_6_-reactive cells four weeks after BCG-vaccination. Red symbols represent the normal T cell- and CD4-CD8-distribution in PBMCs from naïve control guinea pigs. Asterisks indicate the level of significance as determined by unpaired t-test. (**C**) Lymphocytes were stimulated with PIM_6_ as described above. After 24 h RNA was harvested. Cytokine-transcript levels were determined by qRT-PCR in relation to β-Actin. Open bars represent non-stimulated controls, black bars represent transcript levels after PIM_6_-stimulation. The left panel shows the results for BCG-vaccinated animals, the right panel shows the upregulation of IFNγ, GM-CSF and IL17 for naïve, non-immunized animals. Black bars represent the group mean. Error bars indicate the standard error of the mean. Asterisks depict the level of significance as determined by Wilcoxon-matched-pair-signed-ranked-test in comparison to non-stimulated medium controls. (**D**) IFNγ-transcript levels were assessed in the presence of CD1b-blocking or isotype-matched control antibodies. Individually colored circles represent transcript levels of eight BCG-vaccinated guinea pigs four weeks after vaccination. Asterisks depict the level of significance as determined by Wilcoxon-matched-pair-signed-ranked-test as indicated.

**Figure 2:**
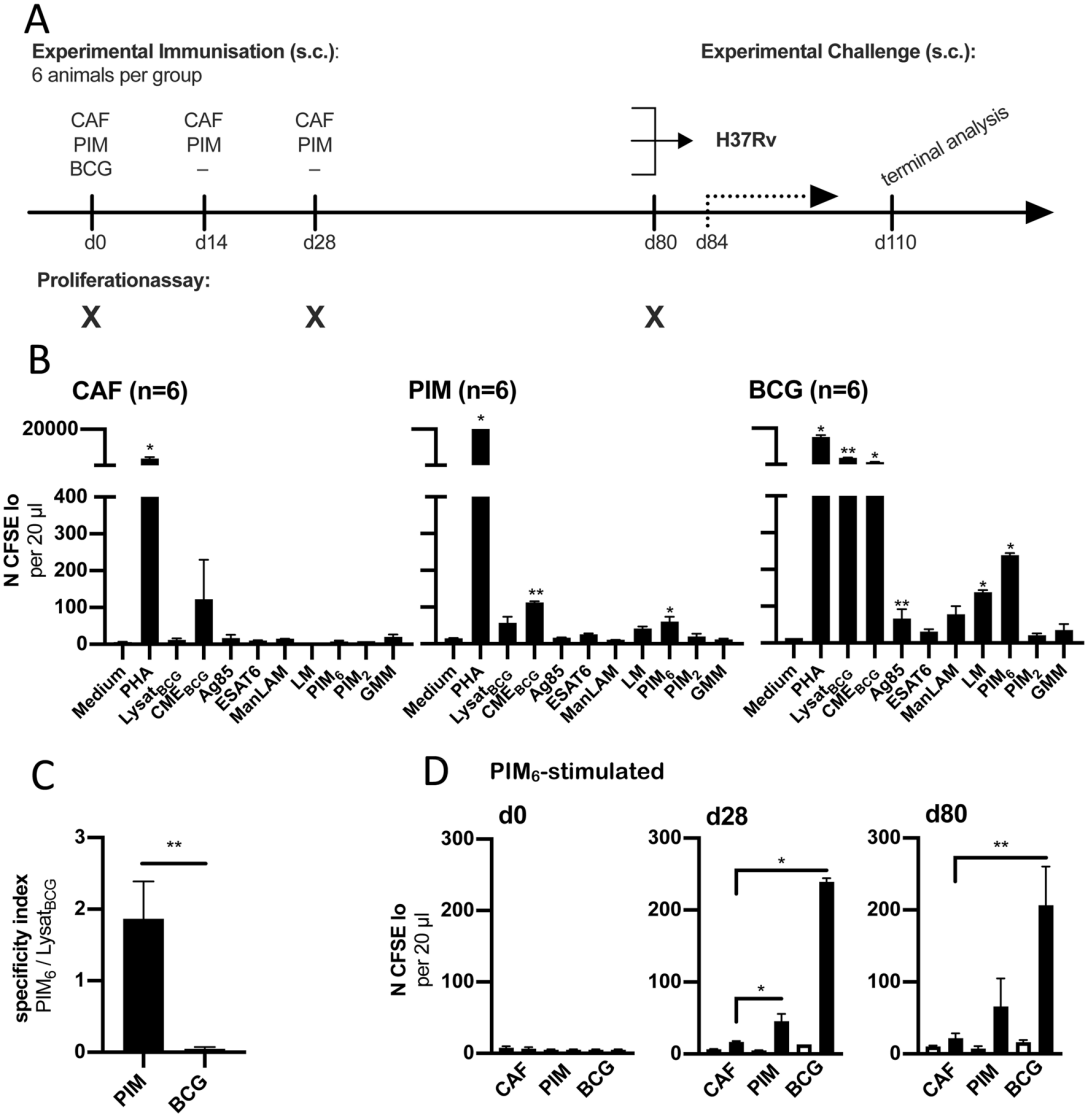
CAF01-adjuvanted PIM_6_ induces PIM_6_-reactive T cell-responses. (**A**) Six guinea pigs per group were vaccinated three times with CAF01-adjuvanted PIM_6_ **[PIM]**, empty CAF01-liposomes **[CAF]** or BCG **[BCG]**. After twelve weeks the animals were challenged with *M. tuberculosis* H37Rv. Four weeks later the animals were euthanized and terminally analyzed. Blood was obtained at the indicated time points. (**B**) 28 days after first vaccination, PBMCs were isolated from vaccinated animals, stimulated and analysed as described for Fig. 1A. Asterisks indicate the level of significance as calculated by nested-t-test in comparison to the medium control. (**C**) The specificity index of the T cell-response 28 days after vaccination was calculated for PIM_6_- and BCG-vaccinated animals by dividing the number of cells that proliferated in response to PIM_6_ by the number of Lysat_BCG_-reactive cells. Asterisks indicate the level of significance as calculated by Mann–Whitney-test. (**D**) The number of PIM_6_-reactive T cells is shown for the different time points tested. Black bars represent the group mean after PIM_6_-stimulation, white bars the respective medium control. Error bars indicate the standard error of the mean, asterisks the level of significance as calculated by nested-t-test in comparison to PIM_6_-stimulated cells from CAF01-vaccinated controls.

**Figure 3:**
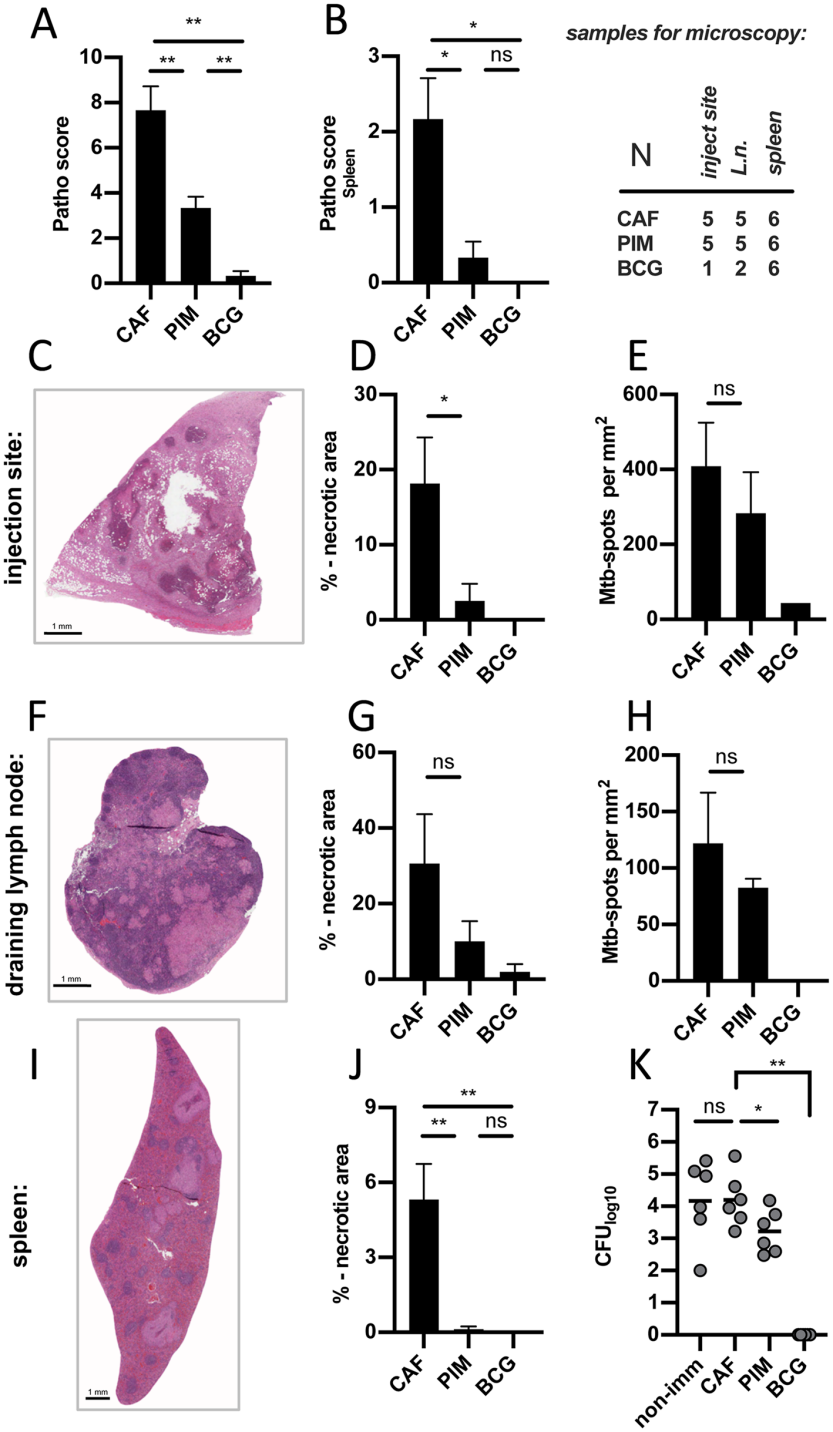
PIM_6_- and BCG-vaccination of guinea pigs is associated with significant reduction in necrosis and bacterial load. Four weeks after s.c. challenge with virulent *Mtb*, the same PIM_6_-vaccinated [**PIM**] and control guinea pigs [**CAF**, **BCG**], six animals per group, were euthanized and necropsied. A gross pathology score shows the overall severity of granulomas as individual sum score (**A**) or focused at the organ level for the spleen (**B**), (n = 6) per group. (**C–J**) Tissue sections of the injection-site-granuloma, the draining lymph node and the spleen were stained with hematoxylin–eosin or immunophenotyped using an *Mtb*-specific antibody. In particular with BCG-vaccinated animals lesions were so small that we could not perform all analyses in parallel. The number of available histological specimen is depicted. The relative area of necrosis within granulomas and the number of *Mtb*-positive spots per mm^2^ were determined for the injection-site-granuloma (**C–E**), the draining right axillary lymph node (**F–H**) and for the spleen (**I**, **J**). Representative sections are shown from CAF01-vaccinated animals. Black bars represent the group mean, error bars represent the standard error of the mean. Spleen homogenates from the three treated groups and from a group of six non-immunized guinea pigs were cultured and the number of colony-forming units are depicted. Circles indicate individual animals, bars represent the group mean. Asterisks indicate the level of significance as calculated by unpaired-t- or Mann–Whitney-test.

**Figure 4:**
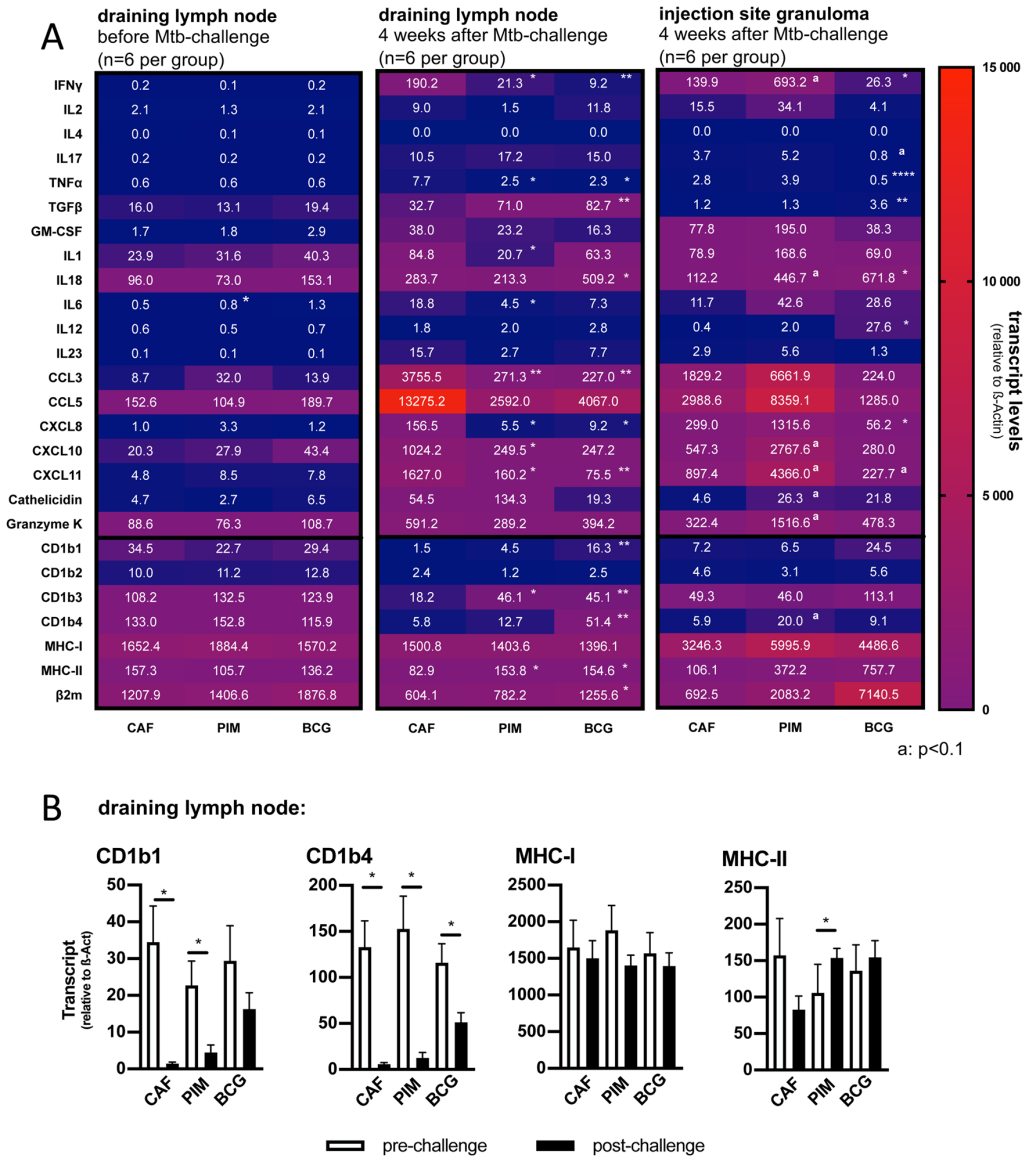
After challenge expression levels of proinflammatory cyto- and chemokines and CD1b-isoforms differ between vaccination groups. Draining lymph nodes of the site of vaccination (6 animals per group) or challenge-inoculation and injection-site-granulomas from *Mtb-*challenged animals (6 animals per group) were harvested and homogenized to isolate bulk RNA. Transcript-levels of the indicated immune genes were quantified by qRT-PCR in relation to β-Actin. (**A**) The left heatmap shows the expression level in the draining lymph node 80 days after vaccination. In the middle the expression level in the draining lymph node, and on the right the injection-site-granuloma four weeks later after virulent *Mtb-*challenge is shown. Columns correspond to respective vaccination groups, rows to the corresponding gene. Expression values are shown as group mean calculated in relation to β-Actin. Asterisks indicate the level of significance, as calculated by Mann–Whitney-test in comparison to CAF01-controls. (**B**) Bar graphs show selected transcript-levels of antigen presenting molecules in the draining axillary lymph node before and four weeks after challenge. Black bars represent the group mean and error bars the standard error of the mean. Asterisks indicate the level of significance as calculated by unpaired-T-test.

**Figure 5:**
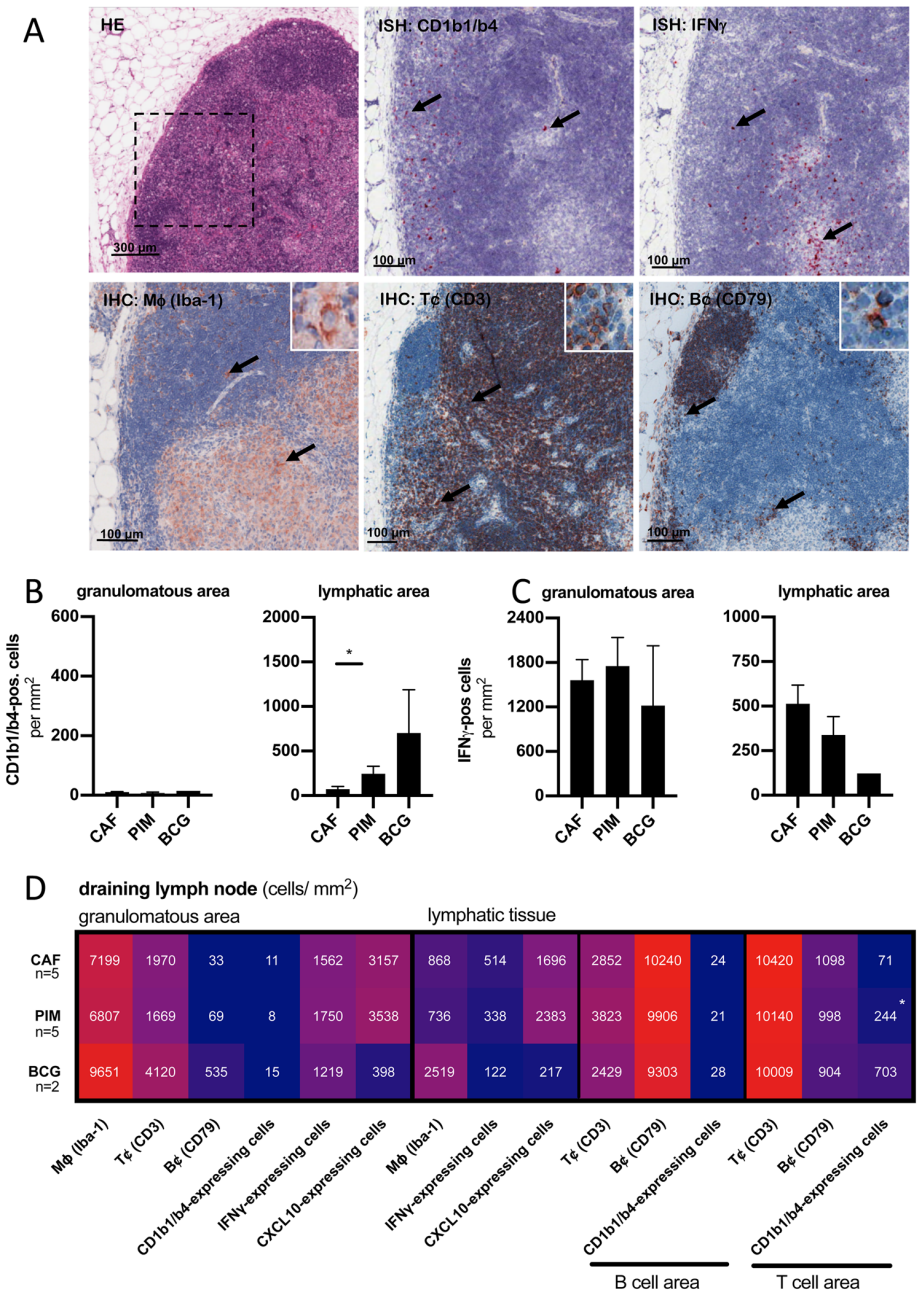
Four weeks after *Mtb*-challenge CD1b1/b4-expressing cells localize in the T cell-rich subcapsular areas of draining lymph nodes. Sections of the same draining lymph nodes as shown before were investigated by ISH and IHC. (**A**) An HE-stained section of the right axillary lymph node from a PIM_6_-vaccinated animal is shown in low magnification. The rectangle is shown in higher magnification for consecutive sections stained by IHC for Iba-1 positive macrophages, CD3-expressing T cells and CD79-expressing B cells (inserts show single positive cells) and by ISH for the expression of CD1b1/b4 and IFNγ. Black arrows indicate single positive cells. The number of CD1b1/b4- (**B**) or IFNγ-expressing (**C**) cells per mm^2^ within lymph node granulomas or the surrounding lymphatic area was quantified. Black bars represent the group mean, error bars the standard error of the mean. (**D**) Consecutive sections of the same samples were additionally stained for macrophage marker Iba-1, B cell-marker CD79 or were subjected to ISH using a CXCL10-specific probe. The heatmap shows a comprehensive overview of the numbers of positive cells per mm^2^ within the granulomatous or the surrounding lymphatic areas. Rows correspond to the three vaccination groups, columns to the tested surface marker or transcript. Numbers represent group means for five CAF01- or PIM_6_-vaccinated and two BCG-vaccinated animals. Asterisks indicate the level of significance as calculated by Mann–Whitney-test in comparison to the CAF01-vaccinated group.

The experiments shown in Fig. 1 were performed with 8 BCG vaccinated guinea pigs and 7 control animals. In Fig. 2 another 18 animals separated into three groups of 6 guinea pigs each were vaccinated either with empty liposomes, liposome-formulated PIM_6_ or BCG and analysed for the cellular response. In Figs. 3, 4 and 5 histological and transcriptional analyses of these 18 guinea pigs after *Mtb*-challenge are shown. To analyse the bacterial burden in the spleen of in entirely naïve animals, in Fig. 3K data of another group of 6 non-vaccinated, challenged animals is shown. The data shown in the left panel of Fig. 4A was obtained with an additional group of 18 guinea pigs that were vaccinated accordingly, but not *Mtb*-challenged.GraphPad Prism version 8.1.0 was used to analyse and visualize the data. Quantitative data are expressed as group means. Error bars indicate the standard error of the mean. Data were tested for normal distribution using Shapiro–Wilk and Kolmogorov–Smirnov test. Results from proliferation-assays were analysed by nested-t-tests. qRT-PCR results of PIM_6_-stimulated cells were analysed with a Wilcoxon matched-pair-signed-rank-test. For the other data shown, the level of significance was calculated by unpaired-t- or Mann–Whitney-U-test. P-values are expressed as follows: *, p < 0.05; **, p < 0.01, ***, p < 0.001 and ****, p < 0.0001. Low-case letter “a” indicates a non-significant tendency (p < 0.1). Data without symbol did not reach significance. To explicitly indicate “non-significance” some data are labelled with “ns”.

## Results

### BCG-vaccination induces robust PIM_6_-specific T cell-responses

PBMCs from BCG-vaccinated or control-animals were isolated four weeks after vaccination and stimulated with mycobacterial antigens in the presence of CD1-expressing, autologous APCs. By flow cytometry, the number of proliferated, CFSE low cells was measured. Cells derived from controls only reacted to phytohaemagglutinin (PHA). Cells from BCG-vaccinated animals vigorously responded to BCG-lysate or CME_BCG_. In addition, there was significant proliferation towards PIM_6_. No proliferation was found in response to PIM_2_, GMM and PDIM (Fig. 1A). The phenotype of proliferating cells was analysed by flow-cytometry. The majority of PIM_6_-specific cells was CD4-positive. A robust percentage of about 20% was CD4-/ CD8- double negative. A minority of proliferating cells was CD8-positive. For comparison, the CD4/ CD8-distribution was assessed in non-stimulated PBMCs from naïve control guinea pigs, shown in red (Fig. 1B). The pattern of CD8-positive and double-negative T cells among PIM_6_-specific, proliferated cells differed significantly from the normal distribution (p = 0.024 and 0.031, resp.). Transcript levels of a selected panel of T cell-cytokines were determined by qRT-PCR. β-Actin served as housekeeping gene. PIM_6_-stimulation induced in almost all cases an upregulation of the Th1-lead cytokine, IFNγ. This was highly significant. Also, the upregulation of GM-CSF and IL17 reached significance. A non-significant increase was found for IL2 transcripts. No upregulation was observed with TNF-α and IL4, while a non-significant reduction was observed with TGF-β. No upregulation of IFNγ and IL17 and only weak and insignificant upregulation of GM-CSF was observed when cells from naïve control animals were stimulated accordingly (Fig. 1C). To analyse the dependence on CD1b-presentation, IFNγ-upregulation was measured after PIM_6_-stimulation in presence of CD1b-blocking antibody. CD1b-blockade significantly reduced upregulation of IFNγ in comparison to the stimulation without antibodies or in the presence of the isotype-control. Two out of eight animals showed a weak reactivity to PIM_6_ and for one animal the isotype control is missing. However, with all animals IFNγ-upregulation was reduced in presence of CD1b-blocking antibody (Fig. 1D).

### CAF01 adjuvanted PIM_6_ induces PIM_6_-specific memory T cell-responses

To investigate the functional role of PIM_6_-specific T cells a vaccination- and challenge-experiment was performed. The experimental outline is shown in Fig. 2A. Three groups of guinea pigs were vaccinated with CAF01-formulated PIM_6_, with BCG or with CAF01 alone. Four weeks after first vaccination, blood was obtained and cell proliferation was tested as described above. Cells from animals that received CAF01 alone only showed significant proliferation after stimulation with PHA. After PIM_6_-vaccination, significant proliferation was observed in response to PIM_6_ and CME_BCG_, which contains high amounts of various PIM-species. BCG-vaccinated animals showed a vigorous response to BCG-lysate and to CME_BCG_. Lower but significant was the proliferation to Ag85, LM and PIM_6_ (Fig. 2B). It has to be emphasized that the absolute level of PIM_6_-reactivity was higher in BCG-vaccinated animals compared to PIM_6_-vaccinated. However, there was a striking difference in the relative responses. In PIM_6_-vaccinated animals the reactivity to BCG-lysate -inducing the strongest response in BCG-vaccinated animals- did not reach significance, only with CME and PIM_6_ a significant reactivity was reached. To illustrate the specificity of the response, the ratio between number of cells that proliferated in response to PIM_6_ and those that proliferated in response to BCG-lysate was separately calculated for PIM_6_- and BCG-vaccinated animals (Fig. 2C). Figure 2D shows the time course of PIM_6_-reactivity for the three vaccination groups. Before vaccination, there was no proliferation to PIM_6_. After 28 days there was a significant response to PIM_6_ in BCG- and PIM_6_-vaccinated animals. After 80 days, four days before *Mtb-*challenge, there was a clear tendency of PIM_6_-reactivity in both groups, but for PIM_6_-vaccinated this did not reach significance. Animals receiving only empty CAF01-liposomes did not react to PIM_6_.

### PIM_6_- and BCG-vaccination of guinea pigs is associated with significant reduction in necrosis and bacterial loads

To assess the role of PIM6-specific T cells, guinea pigs were subcutaneously challenged with virulent *M.tuberculosis.* Although the subcutaneous infection does not recapitulate the natural route of infection, it allowed for a detailed analysis of the interaction between the site of pathogen entry and the draining lymph node. Four weeks after *Mtb-*challenge, granulomas had developed at the site of infection, in dependence on the vaccination group. Granulomas were prominent and widespread in CAF01-controls. Size and distribution of lesions at the site of infection and in the draining lymph node and other organs (e.g. spleen and liver) was assessed macroscopically using a semiquantitative scoring system. PIM_6_- or BCG-vaccinated animals showed a significantly reduced lesion score compared to the CAF01-controls (Fig. 3A). Systemic mycobacterial dissemination was absent or significantly reduced as indicated by normal spleens in BCG-vaccinated, and fewer and smaller granulomas in PIM_6_-vaccinated animals (Fig. 3B). Similar observations were made, when the injection-sites, the draining lymph nodes and spleens were microscopically investigated. Percentage of necrotizing granulomas was significantly reduced in PIM_6_-vaccinated compared to CAF01-controls (Fig. 3C and D, I and J). Only in the lymph node this did not reach significance (Fig. 3F and G). Representative histological sections for all three vaccination groups are shown in supplemental Fig. S1. By immunohistochemistry the number of *Mtb*-specific spots was assessed in injection-site-granulomas and draining lymph nodes. There was a slight reduction in PIM_6_-vaccinated compared to CAF01-controls (Fig. 3E and H). This tendency was confirmed by quantifying the bacterial loads in spleen homogenates: CFUs obtained after challenge in non-immunized, naïve animals were almost identical to CAF01-controls. By contrast, there was a significant one log reduction in the PIM_6_-vaccinated group. In spleens from BCG-vaccinated animals no viable mycobacteria were detected (Fig. 3K).

### After challenge expression levels of proinflammatory cyto- and chemokines and CD1b-isoforms differ between vaccination groups

Transcript levels of several immune genes were determined in the draining lymph node and the injection-site before and four weeks after *Mtb* challenge. Low transcript levels in the draining lymph node were recorded prior to the challenge. Four weeks after challenge, CAF01-controls showed high levels of proinflammatory cyto- and chemokines. By contrast, PIM_6_- and BCG-vaccinated animals expressed significantly lower levels of IFNγ, TNF-α and CCL3, CXCL10 and CXCL11, but elevated levels of TGF-β. At the injection-site, PIM_6_-vaccinated animals showed higher transcript levels of IFNγ, CXCL10, CXCL11, cathelicidin and granzyme K as CAF01-controls. The difference was close to significance (p < 0.1). BCG-vaccinated animals expressed significantly lower levels of IFNγ and TNF-α, but significantly higher levels of TGF-β, IL12 and IL18. After the challenge, there was a dramatic decrease of CD1b-expression, which affected all CD1b-isoforms. The decrease was less pronounced in PIM_6_- and BCG-vaccinated animals, leaving a significant difference to CAF01-controls (Fig. 4A). The discrepancy between transcript levels of CD1b1 before and after challenge was significant for CAF01- and PIM_6_-vaccinated. For CD1b4 the difference was significant in all three groups. No difference before and after challenge was observed for transcript levels of peptide-presenting MHC-I molecules. In contrast to CAF01-vaccinated animals, PIM_6_-vaccinated guinea pigs showed a significant higher level of MHC-II-expression (Fig. 4B).

### CD1b1/b4-expressing cells localize in T cell-rich subcapsular areas of draining axillary lymph nodes

To understand the spatial distribution of different cell populations in the draining lymph node, classical immunohistochemistry was combined with in situ hybridization four weeks after challenge (Fig. 5). CD1b1/b4-expressing cells were almost exclusively found in T cell-areas close to the subcapsular sinus. Only few CD1b1/b4-expressing cells were detected in the macrophage-rich areas of the granuloma. In lymphatic areas of lymph nodes significantly higher numbers of CD1b1/b4-expressing cells were found in PIM_6_- compared to CAF01-vaccinated animals. Numerous CD1b1/b4-expressing cells were observed in lymph nodes of BCG-vaccinated animals, but as only two BCG-samples were available the difference only showed a non-significant tendency (p < 0.1) (Fig. 5B). A representative comparison between CD1b1/b4-expression in the lymph node of a PIM_6_-, a BCG- and a CAF01-vaccinated animal is shown in supplemental Fig. S2. Distribution of IFNγ-expressing cells showed an inverse pattern: The majority of IFNγ-positive cells was found within macrophage-rich areas of granulomas. In lymphatic areas, IFNγ-expression was less abundant. In PIM_6_- and in BCG-vaccinated animals there were fewer IFNγ-expressing cells, although the differences did not reach significance (Fig. 5C). Within lymph node granulomas, activated macrophages represented the predominant cell type, comprising foamy and epitheloid macrophages as identified by their morphological appearance, followed by CD3-positive T- and some scattered B cells (Fig. 5D). In BCG-vaccinated animals T- and B cells were more abundant than in other groups. As a prominent Th1-related chemokine also CXCL10-expression was assessed: In correspondence to transcriptional data obtained by qRT-PCR, a reduction of CXCL10-expressing cells in samples obtained from BCG-vaccinated animals was observed. In lymphatic areas, this finding paralleled the reduced numbers of IFNγ-expressing cells (Fig. 5D).

## Discussion

The role of lipid-reactive T cells in the fine-tuned interplay between pathogens and immune system is still only partially understood. The current study aimed to elucidate whether lipid-reactive T cells contribute to immune protection against virulent mycobacteria. For this, guinea pigs are the ideal model, because, in contrast to murine rodents, they naturally express a functional CD1-group1-system^22^, they are highly susceptible to mycobacterial infections and develop similar pathology as human TB-patients^36^. In the guinea pig model, BCG-vaccination confers relatively robust protection against virulent *Mtb*^37^. In a first step, we therefore tested the initiation of anti-mycobacterial lipid responses by BCG-vaccination. Of a limited set of different lipid antigens only PIM_6_ induced a significant response in BCG-vaccinated guinea pigs. As we used outbred guinea pigs and an indirect stimulation assay to measure PIM_6_-reactivity, the nature of that T cell-response has to be interpreted with caution. However, the fact that prior to immunization no reactivity was observed and also that control animals did not respond to the PIM_6_-preparation precludes the possibility that the observed ex vivo proliferation to PIM_6_ in BCG- or PIM_6_-vaccinated animals was due to innate immune activation, e.g. by the TLR2-agonistic activity of PIMs^38^. The reaction represents a *bona fide* recall-response of T cells that were induced in the course of immunization. BCG-vaccination induced a strong response to the entire breadth of mycobacterial antigens. This is clearly reflected by the strong proliferation to BCG-lysate. Native lipid preparations of mycobacteria are at risk to be contaminated with hydrophobic peptides that can be immunogenic by themselves^4^. We cannot formally exclude the possibility that a certain percentage of the BCG-induced, polyclonal T cell-response to the PIM_6_-preparation reacted to such contaminants. However, results shown in Fig. 1D demonstrate a significant reduction of PIM_6_-induced IFNγ-upregulation in the presence of CD1b-blocking antibodies. This suggests that at least a predominant part of the PIM_6_-reactivity depends on CD1b-presentation. This observation significantly contributes to the ongoing discussion on the role of DURT cells as targets for future TB-vaccines^39^. Very recently, two prospective cohort studies came to the conclusion that BCG vaccination had no effect on CD1b-restricted DURT cells^40^. However, in this study CD1-restricted T cells were only tested by flowcytometry using GMM-loaded CD1-tetramers. In our study BCG-vaccinated guinea pigs also did not respond to GMM. This may indicate that BCG is poorly inducing GMM-specific T cells, but does not necessarily preclude a possible role for CD1-restricted DURT cells.

Despite the limitation of available tools, we tried to characterize PIM_6_-reactive T cells phenotypically. We observed that the majority was CD4-positive, while a substantial minority was CD4-CD8-double-negative. This pattern differed from normal distribution of CD4- and CD8-positive cells. This corresponds to findings in humans, as it has been shown that the majority of lipid-reactive, CD1b-restricted human T cells are CD4-positive^20^. In addition, *bona fide* CD1b-restricted T cell-clones have been described that are double-negative, and it was initially believed that a double-negative phenotype was a marker for CD1-restriction^41,42^.

For specific induction of PIM_6_-reactive cells, we formulated PIM_6_ in CAF01-liposomes. This was a natural choice, because CAFs have been shown to be immunogenic in several animal models and have entered clinical trials^43^. Their efficacy has already been demonstrated in guinea pigs^30^. In a previous study, we formulated PIMs into CAF01, investigated the biophysical properties of resulting liposomes and tested their protective effect against *Mtb*^25^. However, unvaccinated animals served as controls and mechanistical aspects of the immune protection were not addressed. Since the stimulation with empty CAF01-liposomes, containing Mincle-agonist, TDB^44^, could itself induce some immune protection, in the current study, we used animals that received empty CAF01-liposomes as controls. In addition, we investigated the antigen-specific immune response and observed that animals vaccinated with PIM_6_-liposomes mounted a focussed response to PIM_6_. There was no reaction to immunogenic proteins, such as Ag85, and no additional reactivity to the BCG-lysate – beyond the response to PIM_6_. These data are consistent with a model wherein the effect of the PIM_6_-vaccination on the trajectory of the mycobacterial challenge infection, as compared to CAF01-controls, can be attributed to PIM_6_-specific cells. For the current study, we modified a well-established, parenteral challenge protocol^45^. We are aware that this does not recapitulate the natural route of infection, but subcutaneous inoculation induced a very defined primary complex, consisting of the granuloma at the injection site and corresponding lesions in the draining lymph node. This allowed for a detailed analysis of the lymph node response. In accordance with our previous study^25^, we found fewer and smaller necrotizing granulomas in presence of PIM_6_-specific T cells and overall a significantly-reduced bacterial burden. Compared to CAF01-controls, PIM_6_-vaccinated animals showed increased levels of proinflammatory cyto- and chemokines at the site of injection, but reduced levels in the draining lymph node. This may indicate that preformed, PIM_6_-specific T cells contributed to a more efficient control of mycobacteria at the site of infection. Whether the significantly reduced pathology can simply be attributed to an enhanced control of the bacterial growth is not entirely clear at the moment. Compared to BCG-vaccinated animals, PIM_6_-vaccinated still bore a significant bacterial burden. An additional, but not mutually exclusive explanation could lie in the observation that both BCG- and PIM_6_-vaccinated animals showed a different cytokine pattern in the draining lymph node. In particular, BCG-vaccinated animals expressed significantly lower levels of IFNγ and TNFα but elevated levels of TGFβ and IL12. This is in accordance with previous reports^46^. This observation may indicate that -in addition to an improved control of bacterial growth- vaccinated animals were better able to control overshooting inflammatory responses generally causing immune pathology. At the same time, it argues against concerns that CD1b-restricted T cells, as they were investigated in the current study, might aggravate immune pathology through recognition of promiscuous, cross-reactive lipid antigens^47^. The fact that BCG-vaccinated animals were by far better protected than PIM_6_-vaccinated may also be related to the fact that BCG-mediated protection involves additional immune mechanisms related to trained immunity^48^.

To investigate immune processes at the cellular level, we conducted comprehensive microscopic analyses. By ISH we confirmed the reduced expression of IFNγ and CXCL10 in the draining lymph nodes of PIM_6_- and BCG-vaccinated animals. The majority of positive cells resided in the macrophage-rich areas of granulomas. The most likely source of IFNγ were CD3 positive T cells, while CXCL10 was predominantly associated with Iba1-positive macrophages. Important in our context was the distribution of CD1b-expression. High numbers of CD1b1/b4-expressing cells were found at early time points during granuloma development (preliminary data not shown). When granulomas had reached a mature stage, the numbers of CD1b1/b4-expressing cells inside granulomas declined (see Supplemental Figs. S2 and S3). CD1-expression was still detectable, but the majority of granuloma-resident macrophages was not associated with CD1b1/b4-expression. This is in accordance with a study that investigated the presence of CD1b-expressing cells in human TB-granulomas. In this study, it was found that individual cells stained positive for CD1b, while the majority of granuloma-resident macrophages was CD1b-negative^49^. Intriguingly, in CAF01-controls fewer CD1-expressing cells were observed than in PIM_6_- or BCG-vaccinated animals. Maybe, the maintained CD1b-expression in PIM_6_- and BCG-vaccinated was due to a feedback mechanism involving interaction of lipid-reactive T cells with CD1-expressing APCs. A direct back-signaling is unlikely, as no phosphorylation motifs have been described in the cytoplasmic domain of CD1-molecules^50^. However, a cross-talk has been described for CD1d-restricted NKT cells that regulates dendritic cell function and possibly maintains CD1d-expression via a GM-CSF-mediated pathway^51,52^. The close correlation between number of proliferated PIM_6_-specific T cells 28 days after the first immunization and CD1b1- and CD1b4-transcript levels four weeks after challenge points in this direction (see Supplemental Fig. S4A). Another observation in this context was the presence of large numbers of CD1b1/b4-expressing cells in subcapsular areas of draining lymph nodes. This localization is important, because this is, where the lymph from the tributary region enters the lymph node parenchyma. It has been described that mycobacteria-infected macrophages release exosomes from their endosomal compartment that carry high amounts of mycobacterial PIMs- and other mycobacterial lipids^53^. It may be that the subcapsular CD1b-expressing cells take up lymph-borne, exosome-packaged lipids and present them to CD1b-restricted T cells. Hence, the CD1-T cell-axis could represent a vigilance system that responds to lipid antigens in the lymph and recruits additional immune cells to infected tissues. Along this line, we observed a close correlation between number of T cells infiltrating the injection-site-granuloma and number of CD1b1-expressing cells in the lymph node (see Supplemental Fig. S4B).

Altogether, our study reveals the existence of PIM_6_-reactive T cells in guinea pigs after BCG- and liposome-formulated-PIM_6_-vaccination. Our data suggest that this reactivity is at least in part CD1b-restricted. PIM_6_-reactive cells contribute to reduced pathology and bacterial loads and seem to have a beneficial influence on the course of infection after virulent *Mtb*-challenge. This underscores the relevance of mycobacterial, non-protein antigens. Clearly, BCG induced a more robust protection compared to liposomal PIM_6_, but the small population of PIM_6_-specific cells responding to one single, mycobacterial lipid made a significant difference both in terms of pathology and bacterial burden. This shows that the lipid-T cell-axis can provide a complementary layer of immune protection and emphasises that CD1-restricted, DURT cells should be considered in future approaches to develop a vaccine against tuberculosis.

### Supplementary Information

Supplementary Tables.

Supplementary Figures.

## Data Availability

All data analysed for this study are included in this published article and its supplementary information files.

## References

[1] Andersen P, Doherty TM (2005). The success and failure of BCG - implications for a novel tuberculosis vaccine. Nat. Rev. Microbiol..

[2] van Hooij A (2021). BCG-induced immunity profiles in household contacts of leprosy patients differentiate between protection and disease. Vaccine.

[3] Mattow J (2003). Comparative proteome analysis of culture supernatant proteins from virulent *Mycobacterium tuberculosis* H37Rv and attenuated *M. bovis* BCG Copenhagen. Electrophoresis.

[4] Kaufmann E (2016). BCG vaccination induces robust CD4+ T cell responses to *Mycobacterium tuberculosis* complex-specific lipopeptides in Guinea pigs. J. Immunol..

[5] Bastian M, Braun T, Bruns H, Rollinghoff M, Stenger S (2008). Mycobacterial lipopeptides elicit CD4+ CTLs in *Mycobacterium tuberculosis*-infected humans. J. Immunol..

[6] Layre E (2009). Mycolic acids constitute a scaffold for mycobacterial lipid antigens stimulating CD1-restricted T cells. Chem. Biol..

[7] Gilleron M (2004). Diacylated sulfoglycolipids are novel mycobacterial antigens stimulating CD1-restricted T cells during infection with *Mycobacterium tuberculosis*. J. Exp. Med..

[8] Moody DB (2004). T cell activation by lipopeptide antigens. Science.

[9] Sieling PA (1995). CD1-restricted T cell recognition of microbial lipoglycan antigens. Science.

[10] Joosten SA (2019). Harnessing donor unrestricted T-cells for new vaccines against tuberculosis. Vaccine.

[11] Tatituri RV (2013). Recognition of microbial and mammalian phospholipid antigens by NKT cells with diverse TCRs. Proc. Natl. Acad. Sci. U.S.A..

[12] Torina A, Guggino G, La Manna MP, Sireci G (2018). The Janus face of NKT cell function in autoimmunity and infectious diseases. Int. J. Mol. Sci..

[13] Shahine A, Van Rhijn I, Rossjohn J, Moody DB (2023). CD1 displays its own negative regulators. Curr. Opin. Immunol..

[14] de la Salle H (2005). Assistance of microbial glycolipid antigen processing by CD1e. Science.

[15] Kasmar AG (2011). CD1b tetramers bind αβ T cell receptors to identify a mycobacterial glycolipid-reactive T cell repertoire in humans. J. Exp. Med..

[16] Nigou J (1997). The phosphatidyl-myo-inositol anchor of the lipoarabinomannans from *Mycobacterium bovis* bacillus Calmette Guerin. Heterogeneity, structure, and role in the regulation of cytokine secretion. J. Biol. Chem..

[17] Moody DB, Zajonc DM, Wilson IA (2005). Anatomy of CD1-lipid antigen complexes. Nat. Rev. Immunol..

[18] Moody DB, Porcelli SA (2003). Intracellular pathways of CD1 antigen presentation. Nat. Rev. Immunol..

[19] Van Rhijn I (2017). CD1b-mycolic acid tetramers demonstrate T-cell fine specificity for mycobacterial lipid tails. Eur. J. Immunol..

[20] Kasmar AG (2011). CD1b tetramers bind alphabeta T cell receptors to identify a mycobacterial glycolipid-reactive T cell repertoire in humans. J. Exp. Med..

[21] Koch R (1882). Die Aetiologie der Tuberculose. Berliner Klinische Wochenschrift.

[22] Hiromatsu K (2002). Characterization of guinea-pig group 1 CD1 proteins. Immunology.

[23] Dascher CC (1999). Conservation of a CD1 multigene family in the guinea pig. J. Immunol..

[24] Dascher CC (2002). Conservation of CD1 intracellular trafficking patterns between mammalian species. J. Immunol..

[25] Larrouy-Maumus G (2017). Protective efficacy of a lipid antigen vaccine in a guinea pig model of tuberculosis. Vaccine.

[26] Watanabe Y (2006). BCG vaccine elicits both T-cell mediated and humoral immune responses directed against mycobacterial lipid components. Vaccine.

[27] Dascher CC (2003). Immunization with a mycobacterial lipid vaccine improves pulmonary pathology in the guinea pig model of tuberculosis. Int. Immunol..

[28] Hiromatsu K (2002). Induction of CD1-restricted immune responses in guinea pigs by immunization with mycobacterial lipid antigens. J. Immunol..

[29] Agger EM (2008). Cationic liposomes formulated with synthetic mycobacterial cordfactor (CAF01): A versatile adjuvant for vaccines with different immunological requirements. PLoS ONE.

[30] Commandeur S (2014). The in vivo expressed *Mycobacterium tuberculosis* (IVE-TB) antigen Rv2034 induces CD4(+) T-cells that protect against pulmonary infection in HLA-DR transgenic mice and guinea pigs. Vaccine.

[31] Spohr C (2015). A new lymphocyte proliferation assay for potency determination of bovine tuberculin PPDs. Altex.

[32] Mitchison DA (1960). A comparison of the virulence in guinea-pigs of South Indian and British tubercle bacilli. Tubercle.

[33] Jain R (2008). Enhanced and enduring protection against tuberculosis by recombinant BCG-Ag85C and its association with modulation of cytokine profile in lung. PLoS ONE.

[34] Mulisch M, Welsch U (2019). Romeis - Mikroskopische Technik.

[35] Bankhead P (2017). QuPath: Open source software for digital pathology image analysis. Sci. Rep..

[36] Clark S, Hall Y, Williams A (2014). Animal models of tuberculosis: Guinea pigs. Cold Spring Harb. Perspect. Med..

[37] Keyser A, Troudt JM, Taylor JL, Izzo AA (2011). BCG sub-strains induce variable protection against virulent pulmonary *Mycobacterium tuberculosis* infection, with the capacity to drive Th2 immunity. Vaccine.

[38] Gilleron M, Quesniaux VF, Puzo G (2003). Acylation state of the phosphatidylinositol hexamannosides from *Mycobacterium bovis* bacillus Calmette Guerin and *Mycobacterium tuberculosis* H37Rv and its implication in Toll-like receptor response. J. Biol. Chem..

[39] Soma S, Lewinsohn DA, Lewinsohn DM (2021). Donor Unrestricted T Cells: Linking innate and adaptive immunity. Vaccine.

[40] Gela A (2022). Effects of BCG vaccination on donor unrestricted T cells in two prospective cohort studies. EBioMedicine.

[41] Gumperz JE, Brenner MB (2001). CD1-specific T cells in microbial immunity. Curr. Opin. Immunol..

[42] Beckman EM (1994). Recognition of a lipid antigen by CD1-restricted alpha beta+ T cells. Nature.

[43] Christensen D, Korsholm KS, Andersen P, Agger EM (2011). Cationic liposomes as vaccine adjuvants. Expert Rev. Vaccines.

[44] Desel C (2013). The Mincle-activating adjuvant TDB induces MyD88-dependent Th1 and Th17 responses through IL-1R signaling. PLoS ONE.

[45] Balasubramanian V, Guo-Zhi W, Wiegeshaus E, Smith D (1992). Virulence of *Mycobacterium tuberculosis* for guinea pigs: A quantitative modification of the assay developed by Mitchison. Tuber Lung Dis..

[46] Ly LH, Russell MI, McMurray DN (2008). Cytokine profiles in primary and secondary pulmonary granulomas of Guinea pigs with tuberculosis. Am. J. Respir. Cell Mol. Biol..

[47] Van Rhijn I (2016). Human autoreactive T cells recognize CD1b and phospholipids. Proc. Natl. Acad. Sci. U.S.A..

[48] Kaufmann E (2018). BCG educates hematopoietic stem cells to generate protective innate immunity against tuberculosis. Cell.

[49] Chancellor A (2017). CD1b-restricted GEM T cell responses are modulated by *Mycobacterium tuberculosis* mycolic acid meromycolate chains. Proc. Natl. Acad. Sci. U.S.A..

[50] Brigl M, Brenner MB (2004). CD1: Antigen presentation and T cell function. Annu. Rev. Immunol..

[51] Racke FK, Clare-Salzer M, Wilson SB (2002). Control of myeloid dendritic cell differentiation and function by CD1d-restricted (NK) T cells. Front. Biosci..

[52] Gillessen S (2003). CD1d-restricted T cells regulate dendritic cell function and antitumor immunity in a granulocyte-macrophage colony-stimulating factor-dependent fashion. Proc. Natl. Acad. Sci. U.S.A..

[53] Beatty WL (2000). Trafficking and release of mycobacterial lipids from infected macrophages. Traffic.

