# Author Correction: Phosphatidylinositolmannoside vaccination induces lipid-specific Th1-responses and partially protects guinea pigs from *Mycobacterium tuberculosis* challenge

**DOI:** 10.1038/s41598-023-49215-w

**Published:** 2023-12-18

**Authors:** Emmelie Eckhardt, Jan Schinköthe, Marcel Gischke, Julia Sehl-Ewert, Björn Corleis, Anca Dorhoi, Jens Teifke, Dirk Albrecht, Annemieke Geluk, Martine Gilleron, Max Bastian

**Affiliations:** 1 Friedrich-Loefer-Institut, Südufer 10, 17493 Greifswald – Isle of Riems, Germany; 2 Institute of Veterinary Pathology, Faculty of Veterinary Medicine, Leipzig University, Leipzig, Germany; 3 Institute of Microbiology, Greifswald University, Greifswald, Germany; 4 Department of Infectious Diseases, Leiden University Medical Center, Leiden, The Netherlands; 5 CNRS, Institut de Pharmacologie et de Biologie Structurale, Toulouse, France

Correction to: *Scientific Reports* 10.1038/s41598-023-45898-3, published online 30 October 2023

The Funding section in the original version of this Article was incomplete.

“Open Access funding enabled and organized by Projekt DEAL.”

now reads:

“Open Access funding enabled and organized by Projekt DEAL. This work was supported by the German Research Foundation (BA 3885/2-1) and the European Commission (H2020-PHC-643381).”

The original Article has been corrected.

