# Supplementary material for: Phosphatidylinositolmannoside vaccination induces lipid-specific Th1-responses and partially protects guinea pigs from Mycobacterium tuberculosis challenge
Source: Sci Rep. 2023 Oct 30;13:18613. doi: 10.1038/s41598-023-45898-3 (PMC10616071; doi:10.1038/s41598-023-45898-3)
Supplement: Supplementary file 1 — Supplementary Tables. [file 41598_2023_45898_MOESM1_ESM.docx]

Supplemental Table 1 – Pathology Score adapted from (27, 28)

| **Parameters** | **Score used** |
| --- | --- |
| **Inoculation site granuloma (right axillary subcutis)** | |
| Granuloma(s) ≥3.5 cm in diameter with caseous necrosis | 4 |
| Granuloma(s) ≥2.5 cm in diameter with caseous necrosis | 3 |
| Granuloma(s) ≥1.5 cm in diameter ± caseous necrosis | 2 |
| Granuloma(s) ≥0.5 cm in diameter | 1 |
| No lesion | 0 |
| **Right axillary lymph node** | |
| Granuloma(s) ≥2.0 cm in diameter with caseous necrosis | 4 |
| Granuloma(s) ≥1.5 cm in diameter with caseous necrosis | 3 |
| Granuloma(s) ≥1.0 cm in diameter ± caseous necrosis | 2 |
| Granuloma(s) ≥0.5 cm in diameter | 1 |
| No lesion respectively of normal size (≤0.5 cm) | 0 |
| **Spleen*** | |
| Numerous granulomas (miliary type) | 4 |
| Many granulomas | 3 |
| Few granulomas | 2 |
| Single granulomas | 1 |
| No lesion | 0 |
| **Liver*** | |
| Numerous granulomas (miliary type) | 4 |
| Many granulomas | 3 |
| Few granulomas | 2 |
| Single granulomas | 1 |
| No lesion | 0 |

*granulomas in spleen and liver were randomly distributed and 0.3 - 0.4 cm in diameter large

Supplemental Table 2 – qRT-PCR primer

| gene | FORWARD-Primer | REVERSE-Primer | Annealing T° | amplicon size |
| --- | --- | --- | --- | --- |
| ifng | ATTTCGGTCAATGACGAGCAT | GTTTCCTCTGGTTCGGTGACA | 60 °C | 90 bp |
| il2 | GCAGTGCACCTACTTCAAGC | ACGCCTTCCAAGAGTGTCTG | 60 °C | 88 bp |
| il4 | TCACGGACGTCTTTGCTGAT | CTCCCTCTCTGTTTGGGCAG | 60 °C | 120 bp |
| il17 | CCTGGGACGCCTTCTTCAAT | TGGACACCCTGGATTTCGTG | 60 °C | 99 bp |
| tnfa | ACGCTCACACTCAGATCAGC | GCTGGTTTGCCACAACATGA | 60 °C | 70 bp |
| tgfb | GCTGCGAATGCAGAGACTCA | AGGTAGCGCCAGGAATTGTT | 61 °C | 84 bp |
| gm-csf | GAGGGCTCCTTGACCTTGATG | ATACAGGAAGTTTCCGGGGTCG | 60 °C | 70 bp |
| il1 | GTTTCAGGCAGACCGTCTCA | GGAAGCAAGGGTCTCAGGTC | 60 °C | 106 bp |
| il18 | GACTCCGACTGTGCAGACAA | CCCGTTACACGTCACAGAGA | 60 °C | 108 bp |
| il6 | TTCCTCTCCACAAGCACCTTC | TATCAGCTGTGAAGTCGTGCT | 60 °C | 106 bp |
| il12p35 | AAAACCAGCACCGTGAAAGC | GGCAACTCCCATTGGTTGTG | 60 °C | 100 bp |
| il23 | GACGTTGATCAGCGACTCCC | CAGCCATCCCCACATAGGAT | 60 °C | 76 bp |
| ccl3 | CCACGTGCATACGTAGCTGA | CTCCCGGCCTCTCTTGGTTA | 60 °C | 87 bp |
| ccl5 | CCGCACCCACATCAAGGAAT | ACACACCTGGCGGTTCTTTC | 61 °C | 91 bp |
| cxcl8 | GGCAGCCTTCCTGCTCTCT | CAGCTCCGAGACCAACTTTGT | 61 °C | 65 bp |
| cxcl10 | CTCTGAGTGGGACTCAAGGAAT | AGGACTTTGGATTAACAGGTTGAGT | 59 °C | 90 bp |
| cxcl11 | ACAGTTGTTCAAGGCTTCCC | GGCTTTTGCAATATCTGCCACTT | 58 °C | 96 bp |
| cathelicidin | AACGAAAACCTCTTCCGCCT | GCTTCGGACTATACGGGTCG | 60 °C | 73 bp |
| grzK | CCAGTCCGACAGCAATCACA | GCTGGCCTTCGACAAACAAAA | 60 °C | 98 bp |
| cd1b1 | GCTTGGTAGCTTTGACAGCG | ACCTCTTCATTGCTGAAGTTTGC | 60 °C | 81 bp |
| cd1b2 | TGCCTTTCCGGAGCCAATTT | GCTTGATTTTGTGCCCAGGT | 61 °C | 78 bp |
| cd1b3 | ACTCAGGATGCCTTCCAAGAA | CTCCCAACCAGCCTGAGAGTT | 59 °C | 103 bp |
| cd1b4 | GTGGATGCGGGGTGATAAGG | ATGTCTCATCAGCATTGGGCA | 61 °C | 71 bp |
| mhc-i | GAGAACGGGAAGGAGACGC | GGGTGACCTGTTCCTTGGAG | 60 °C | 85 bp |
| mhc-ii | TACCTGCCTTTCGTGCCATC | GGATGGGGCTTCGATCTTCA | 60 °C | 112 bp |
| b2m | TGCCTTATGCATCCAGTAAGAAAA | TGGAAGGGCAAACATGCAGA | 59 °C | 100 bp |
| β-act | ATGACGATATCGCTGCGCTC | CCATGCCTACCATGACTCCC | 60 °C | 138 bp |

Primer for the indicated guinea pig genes were designed using the NCBI Primer-BLAST tool to span an amplicon size between 60 and 140 bp.

Supplemental Table 3 – antibodies

| Antibody | Type | Antigen | Host | Dilution | Duration | AR | Temperature | Supplier | Article number |
| --- | --- | --- | --- | --- | --- | --- | --- | --- | --- |
| CD79α | HM57 | CD79 α | mouse | 1:50 | Overnight | HTAR^a^ | 4°C | LSBio Sciences | LS-B4504 |
| CD3 | Polyclonal | CD3 | rabbit | 1:100 | Overnight | HTAR^a^ | 4°C | Agilent | A045229-2 |
| Iba-1 | Polyclonal | Iba1 Fusion Protein Ag1363 | rabbit | 1:500 | Overnight | HTAR^b^ | 4°C | Proteintech | 10904-1-AP |
| *M. tuberculosis* | Polyclonal | PPD from *M. tuberculosis* | rabbit | 1:500 | Overnight | HTAR^b^ | 4°C | BioRad | OBT0974 |
|  |  |  |  |  |  |  |  |  |  |

| a: HTAR: High-temperature antigen retrieval solution (20 minutes at 115°C in TRIS/EDTA-buffer, pH 9) |
| --- |
| b: HTAR: High-temperature antigen retrieval solution (20 minutes at 115°C in citrate buffer, pH 6) |
| Abbreviations: AR: Antigen retrieval; Iba-1: allograft inflammatory factor 1; PPD: Purified protein derivative |
