# Supplementary material for: Phosphatidylinositolmannoside vaccination induces lipid-specific Th1-responses and partially protects guinea pigs from Mycobacterium tuberculosis challenge
Source: Sci Rep. 2023 Oct 30;13:18613. doi: 10.1038/s41598-023-45898-3 (PMC10616071; doi:10.1038/s41598-023-45898-3)
Supplement: Supplementary file 2 — Supplementary Figures. [file 41598_2023_45898_MOESM2_ESM.pdf]

Figure S1

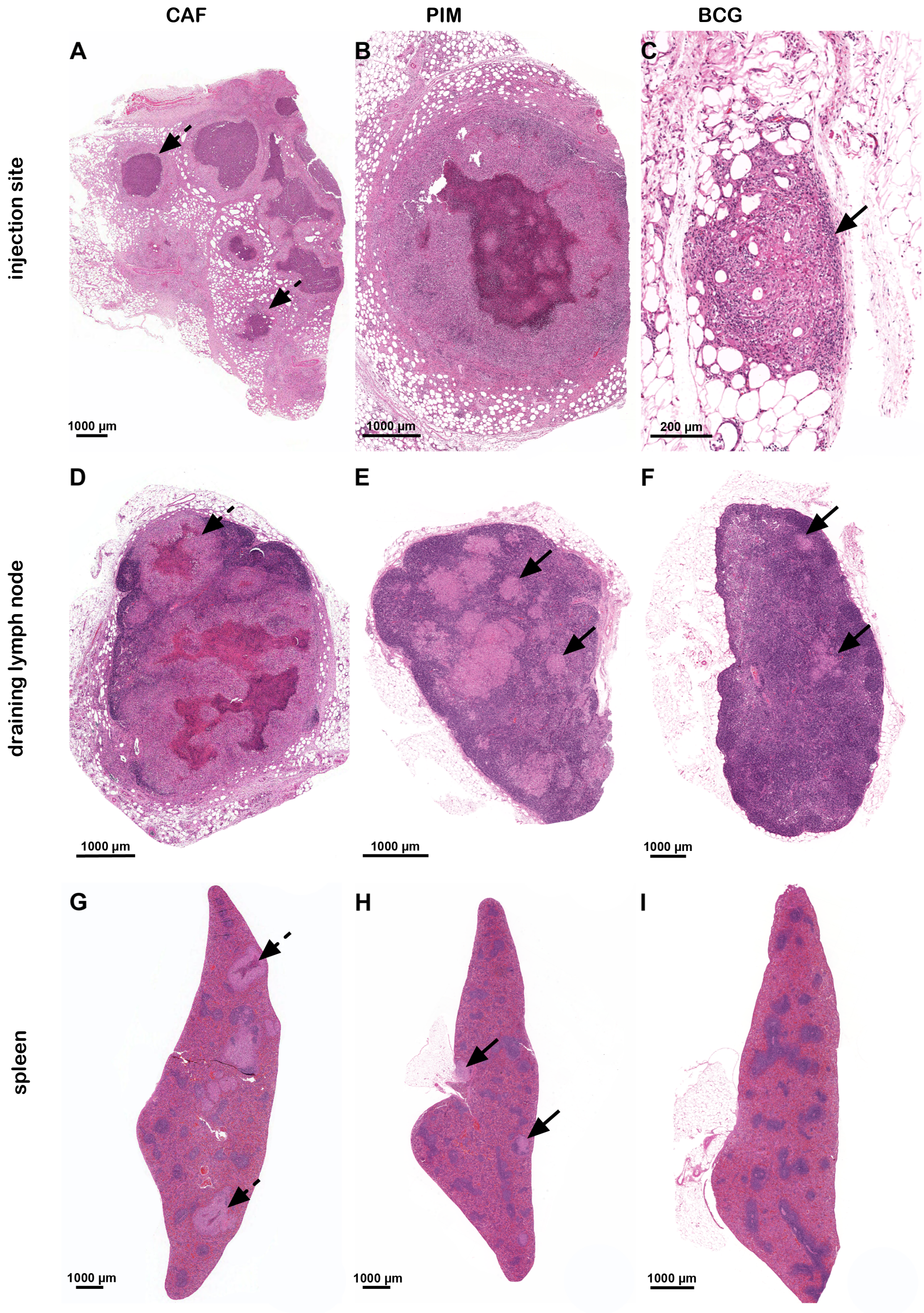
CAF
PIM
BCG
injection site
draining lymph node
spleen
A
B
C
D
E
F
G
H
I
1000 µm
1000 µm
200 µm
1000 µm
1000 µm
1000 µm
1000 µm
1000 µm
1000 µm

Figure S1 – Representative images of granulomas at the injection site, in the draining axillary lymph node and the spleen

Four weeks after s.c. challenge with virulent *Mtb*, PIM_6-, CAF01-, and BCG-vaccinated guinea pigs developed granulomas at the injection site, in the draining right axillary lymph node and spleen. Tissue sections were stained with hematoxylin and eosin and scanned as digital image. Granulomas of varying size and features developed in each subcutis, lymph node or spleen.

(A) Multiple granulomas with extensive necrosis (dashed arrows) are present at the injection site of this CAF01-vaccinated animal. (B) In this PIM_6-vaccinated animal similar granulomas are seen, but the necrosis is smaller. (C) In this BCG-vaccinated animal granulomas are reduced in size and are non-necrotic (arrow).

(D) Up to 90% of the lymph node in this CAF01-vaccinated animal is replaced by multiple granulomas with extensive necrosis (dashed arrow). (E) In this PIM_6-vaccinated animal multiple smaller non-necrotic granulomas (arrows) and fewer larger necrotic granulomas are seen. (F) Only few non-necrotic granulomas are detected in the paracortex of this lymph node from a BCG-vaccinated guinea pig.

(G) The spleen of this CAF01-vaccinated guinea pig shows multiple granulomas, mostly in association with the periarteriolar lymphoid sheaths (PALS) of the white pulp, some of them are of the necrotic type (dashed arrow). (H) Few non-necrotic granulomas (arrows) are seen near the PALS in this PIM6-vaccinated animal. (I) No granulomas are present in this the spleen of this BCG-vaccinated animal.

Figure S2

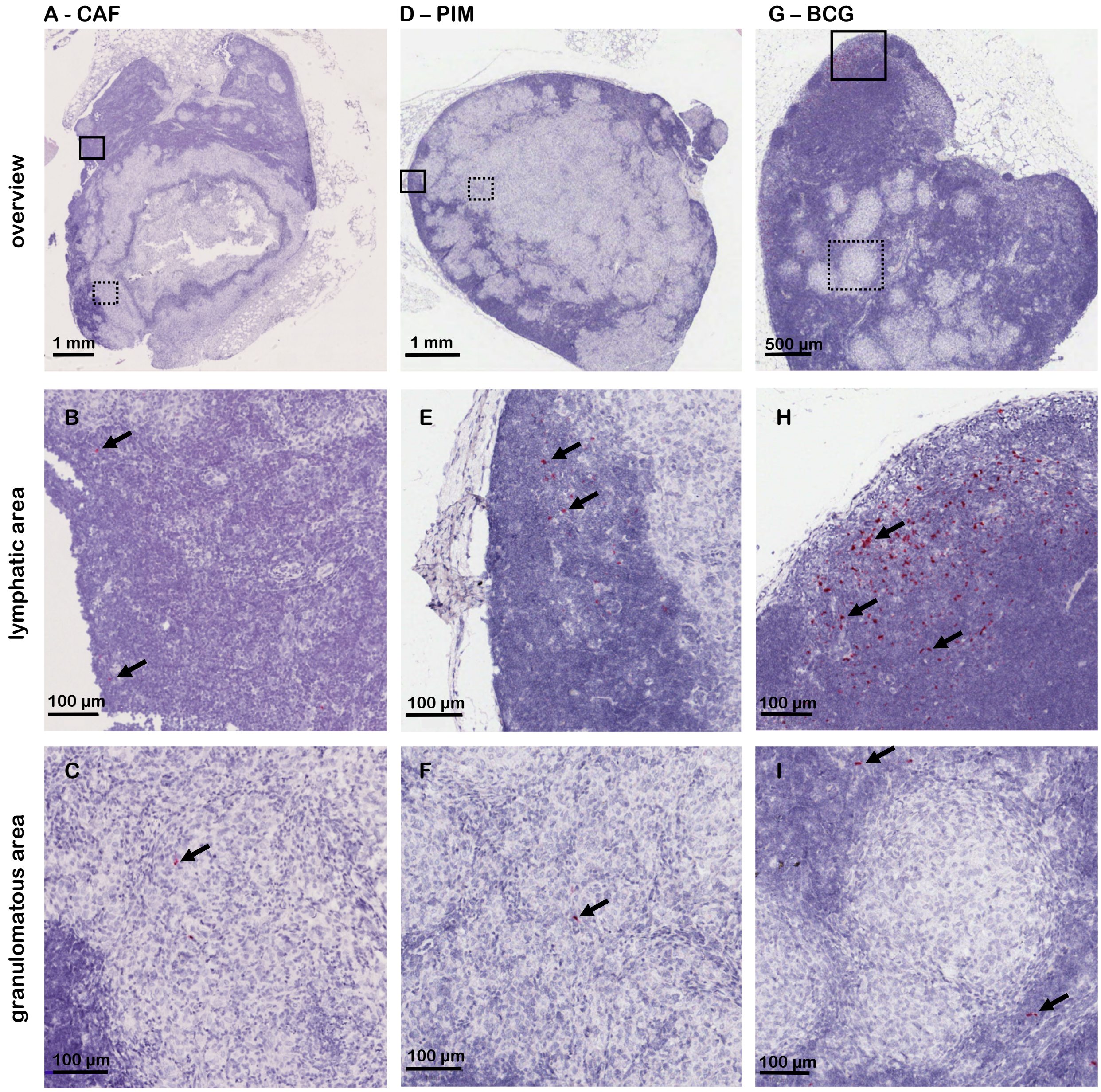
A - CAF
D – PIM
G – BCG
overview
lymphatic area
granulomatous area
1 mm
1 mm
500 µm
B
E
H
100 µm
100 µm
100 µm
C
F
I
100 µm
100 µm
100 µm

Figure S2 –CD1b1/b4-expressing cells are mainly found in the subcapsular region of the draining lymph nodes of PIM_6- or BCG-vaccinated animals

Four weeks after s.c. challenge with *Mtb*, PIM_6-vaccinated [PIM] and control guinea pigs [CAF, BCG] were euthanized and necropsied. FFPE-sections of the draining lymph nodes were investigated by in situ hybridization (ISH) for the expression of CD1b1/b4 (red). Hematoxylin (blue) was used for counterstaining. A-C: In this lymph node of a CAF01-vaccinated guinea pig an extensive necrotic granuloma formation is seen (A). Only few CD1b1/b4-expressing cells are detected in the lymphatic area (B, solid rectangle) and in the macrophage-rich rim of the granuloma (C, dotted rectangle). D-F: Multiple, non-necrotic granulomas deface the axillary lymph node of this PIM_6-vaccinated animal (D). Several CD1b1-expressing cells populate the subcapsular lymphatic area of the lymph node (E, solid rectangle). Only few, individual CD1b1/b4-expressing cells are detected in the macrophage-rich zone of the granuloma (F, dotted rectangle). G-I: In a BCG-vaccinated guinea pig the draining lymph node shows few, non-coalescing, non-necrotic granulomas (G). Numerous CD1b1/b4-expressing cells are present in the subcapsular lymphatic area (H, solid rectangle), no CD1b1/4-expressing cells are detected in the macrophage-rich area of a granuloma (I, dotted rectangle). Arrows indicate CD1b1/b4- expressing cells.

Figure S3

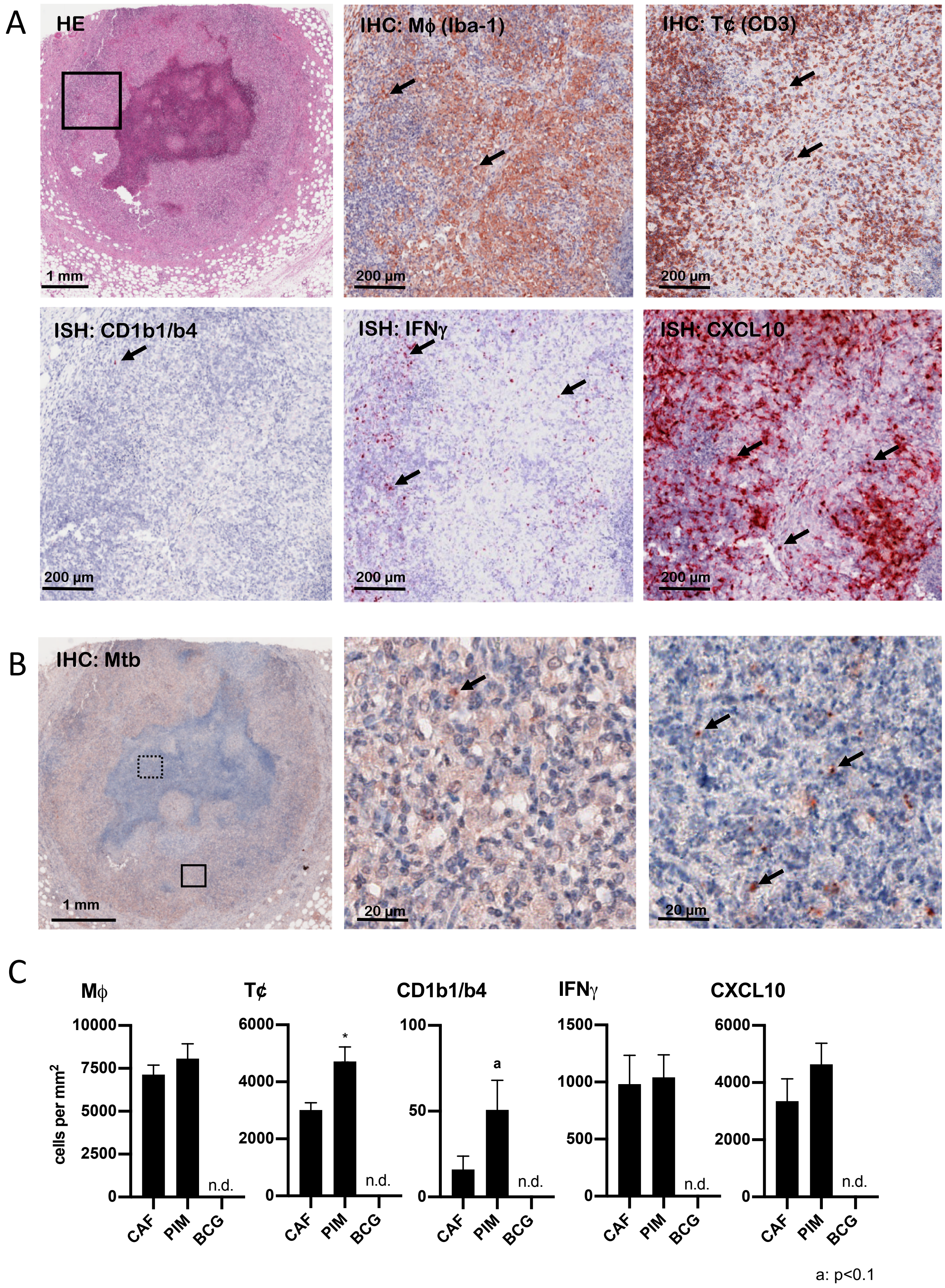
A
HE
1 mm
IHC: Mϕ (Iba-1)
200 μm
IHC: T¢ (CD3)
200 μm
ISH: CD1b1/b4
200 μm
ISH: IFNγ
200 μm
ISH: CXCL10
200 μm
B
IHC: Mtb
1 mm
20 μm
20 μm
C
Mϕ
T¢
CD1b1/b4
IFNγ
CXCL10
cells per mm2
10000
7500
5000
2500
0
6000
4000
2000
0
100
50
0
1500
1000
500
0
6000
4000
2000
0
*
a
n.d.
CAF
PIM
BCG
a: p<0.1

Figure S3 – Comprehensive pathohistological analysis of an injection site granuloma

Four weeks after challenge, sections of the injection site granuloma were investigated by in situ hybridization (ISH) and immunohistochemistry (IHC). (A) Representative stainings of a granuloma from a PIM_6-vaccinated animal are shown: The HE staining provides an overview, the black quadrant is shown in higher magnification for the specific stainings: In the upper panel from left to right: IHC: Iba-1-antibody, IHC: CD3-antibody; in the lower panel from left to right: ISH: CD1b1/4-probe, ISH: IFNγ-probe, ISH: CXCL-10-probe. (B) Consecutive FFPE-sections of the same granuloma were immuno-stained with an antibody specific for *M. tuberculosis.* The micrograph on the left gives an overview, the solid rectangle delineates a macrophage-rich area, which is shown in higher magnification in the middle graph. Few Mtb-specific spots are identified (black arrows). The dotted rectangle delineates part of the necrotic area, which is shown in higher magnification on the right. Several antibody-positive Mtb-spots are indicated by black arrows. (C) The number of Iba-1-positive macrophages, CD3-positive T cells and CD1b1/4-, IFNγ– and CXCL-10-expressing cells in the injection site granuloma was quantified by WSI analysis for PIM_6- and mock-vaccinated animals. Black bars represent the group mean, error bars the standard error of the mean. Asterisks indicate the level of significance as calculated by Mann-Whitney test in comparison to the CAF01-vaccinated group.

Figure S4

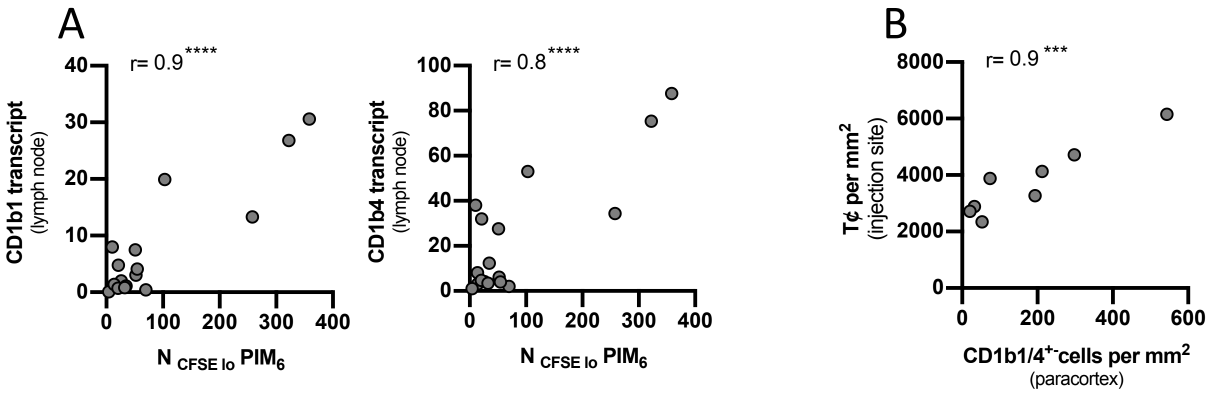

Figure S4– Correlation analysis

Four weeks after s.c. challenge with virulent H37Rv, PIM_6-vaccinated [PIM] and control guinea pigs [CAF, BCG] were euthanized and dissected. (A) The transcript levels of CD1b1 and CD1b4 in the draining lymph node four weeks after virulent H37Rv challenge were quantified by qRT-PCR in relation to β-Actin. The number of T cells proliferating in response to PIM_6 28 days after the first vaccination ($N_{CFSE\ lo}\ PIM_6$) was quantified by flow cytometry. Data was available for all animals from all groups (n=18). The Pearson correlation coefficient between the number of PIM_6-specific T cells and the CD1b expression level four weeks after the challenge was calculated and is indicated (r). (B) Four weeks after the challenge, FFPE-sections of the draining lymph nodes were investigated by in situ hybridization (ISH) for the expression of CD1b1/4 and quantified by WSI analysis. From the same animals FFPE-sections of the injection site granuloma were stained with a CD3-specific antibody. The number of granuloma infiltrating T cells was quantified by WSI analysis. Data from five PIM_6-vaccinated and five CAF-vaccinated control animals were available. The Pearson correlation coefficient was calculated between the number of T cells in the injection site granuloma and the number of CD1b1/4-expressing cells in the paracortex of the draining lymph node and is indicated (r). Grey circles represent individual animals. Asterisks indicate the level of significance.

Figure S5

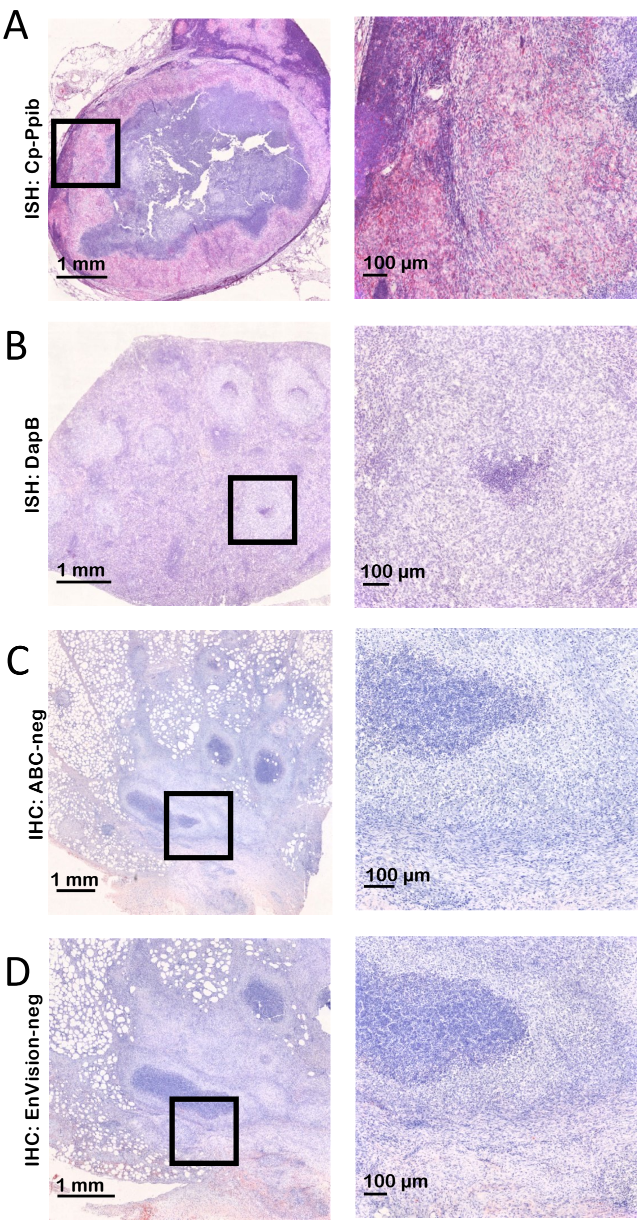

Figure S5 – Representative micrographs of control slides

Four weeks after challenge, sections were investigated by *in situ* hybridization (ISH) and immunohistochemistry (IHC). Negative and positive control slides were made to validate each staining. For every staining the left micrograph gives an overview. The black frames delineate a visual field that is shown in higher magnification on the right. (A) Probe Cp-Ppib was used as positive control for ISH. (B) Probe DapB was used as a negative control for ISH. (C) The ABC-system was used without primary antibody as a negative control for CD79-staining. (D) EnVision-system was used without a primary antibody as a negative control for CD3-, Iba-1- and *Mtb*-staining.

Figure S6

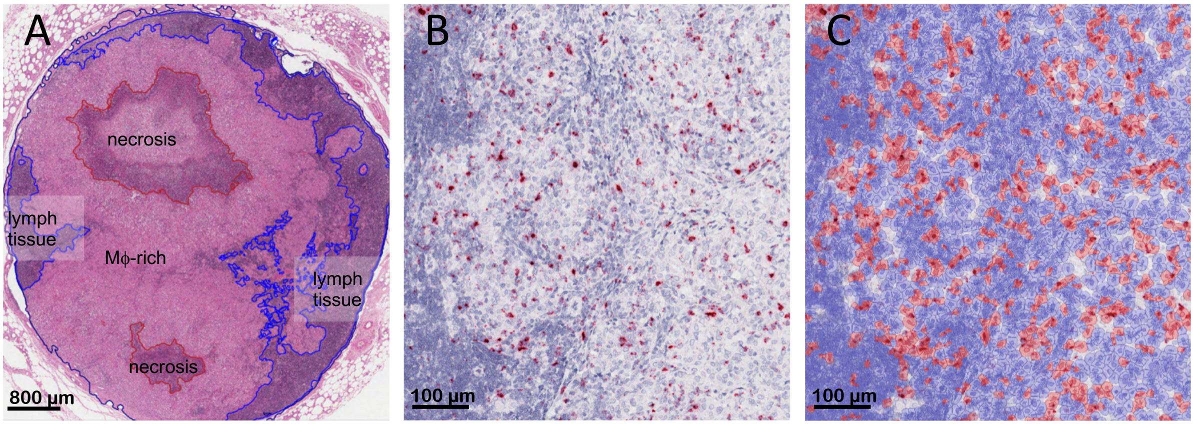

lymph node – 29 mm^2
lymph tissue – 6 mm^2
Mϕ-rich granulomatous area – 18 mm^2
necrotic area – 3 mm^2

analysed area – 1 mm^2
cells identified– 8653
FastRed-positive cells – 1220

Figure S6 – Representative micrographs depicting digital Whole Slide Image (WSI) analysis

Four weeks after challenge, tissue sections were investigated by *in situ* hybridization (ISH) and immunohistochemistry (IHC). Whole slides were scanned and analyzed using QuPath Software.

(A) In HE-stained sections regions of interest (ROIs) were defined as follows: necrotic areas were identified as dark blueish regions comprised of cell debris and necrotic granulocytes (red line). The necrotic areas were differentiated from regions that were dominated by macrophages with large, foamy cytoplasm (Mϕ-rich) and those that contained mostly lymphocytes (lymph tissue, blue line). The ROIs were circumscribed using the *wand* tool of the software and the size was measured. As an example the draining lymph node of a PIM_6-vaccinated guinea pig is shown. (B) To quantify the number of ISH- or IHC-positive cells, cells were automatically identified using the *positive cell selection* tool. The same settings were used to analyze the respective sections from all three groups. As an example, the lymph node of a PIM-vaccinated guinea pig is shown after IFNγ–specific ISH. In (B) the section is shown as scanned, in (C) the same sector is shown after digitally identifying FastRed-positive cells (negative cells shown in blue, positive cells shown in red).
